# Recent Advances in Anti-Inflammatory Compounds from Marine Microorganisms

**DOI:** 10.3390/md22090424

**Published:** 2024-09-18

**Authors:** Guihua Yang, Miaoping Lin, Kumaravel Kaliaperumal, Yaqi Lu, Xin Qi, Xiaodong Jiang, Xinya Xu, Chenghai Gao, Yonghong Liu, Xiaowei Luo

**Affiliations:** 1 Guangxi Key Laboratory of Marine Drugs, Institute of Marine Drugs, Guangxi University of Chinese Medicine, Nanning 530200, China; 2 Unit of Biomaterials Research, Department of Orthodontics, Saveetha Dental College and Hospitals, Saveetha Institute of Medical and Technical Sciences (SIMATS), Saveetha University, Chennai 600077, India

**Keywords:** marine microorganisms, secondary metabolites, chemical structures, anti-inflammatory compounds, structure–activity relationship

## Abstract

Marine microbial secondary metabolites with diversified structures have been found as promising sources of anti-inflammatory lead compounds. This review summarizes the sources, chemical structures, and pharmacological properties of anti-inflammatory natural products reported from marine microorganisms in the past three years (2021–2023). Approximately 252 anti-inflammatory compounds, including 129 new ones, were predominantly obtained from marine fungi and they are structurally divided into polyketides (51.2%), terpenoids (21.0%), alkaloids (18.7%), amides or peptides (4.8%), and steroids (4.3%). This review will shed light on the development of marine microbial secondary metabolites as potential anti-inflammatory lead compounds with promising clinical applications in human health.

## 1. Introduction

Inflammation is a defense reaction caused when the organism is subjected to certain stimuli, such as trauma and infection, which is characterized by malfunction, heat, redness, swelling, and discomfort. Both the natural defense system and inflammatory response have certain advantages for the body. Nevertheless, an excessive inflammatory response tends to damage the tissues of the organism, leading to the development or rapid deterioration of disease [1,2]. If untreated, this may lead to autoimmune or autoimmune inflammatory diseases, neurodegenerative diseases, or even cancer. A series of studies have indicated that inflammation alters the brain’s neurotransmitter systems, which in turn modifies motivation-related behaviors and eventually results in a loss of pleasure [3,4,5]. Inflammation is a key barrier to the treatment of depression and other related mental diseases. It is a typical symptom of mood and anxiety disorders in psychiatric and medical conditions. Currently, the commonly used anti-inflammatory drugs in clinical practice are steroidal and non-steroidal compounds, such as indomethacin, aspirin, prednisolone, dexamethasone, and hydrocortisone [6,7,8]. Suppressing various related factors shows anti-inflammatory effects, but long-term use can produce various side effects, such as edema and gastrointestinal ulcers [9]. Hence, there is an urgent need to search for structurally new and highly effective anti-inflammatory drugs with low toxicity.

Marine microorganisms are exposed to special living environments of high pressure, dark conditions, high salinity, and a low concentration of oxygen [10]. For better adaptation to this special environment, marine microorganisms have evolved unique metabolic pathways and can produce diverse bioactive metabolites [11]. Marine microorganisms, especially marine fungi, have elicited increasing interest from the marine natural product research community [12,13,14,15,16]. Moreover, a series of structurally diverse secondary metabolites with anti-inflammatory activity have been obtained from marine microorganisms, including peptides, polyketides, phenols, lactones, alkaloids, steroids, and others [17,18,19]. Among them, cacospongionolide B and petrosaspongiolide M are two representative examples of anti-inflammatory compounds in experimental models of acute or chronic inflammation [20]. It is anticipated that marine microbial natural products would play a promising role in the search for anti-inflammatory lead compounds [21].

In the previous literature, Xu et al. reviewed 133 marine fungi-derived anti-inflammatory compounds in the period from 2000 to 2018, including alkaloids, terpenoids, polyketides, peptides, and others [22]. Souza Cássio, R.M. et al. summarized 41 marine alkaloids with anti-inflammatory activity and gave future perspectives for their investigation and bioprospecting [23]. Since marine microorganisms have been continuously evidenced as rich sources of anti-inflammatory compounds in recent years, this review summarizes the sources, chemical structures, and pharmacological properties of anti-inflammatory natural products recently reported from marine microorganisms during 2021–2023. A total of 252 compounds with anti-inflammatory activity were obtained from marine microorganisms during 2021–2023, including 129 new ones (51.2%). They were mainly isolated from marine fungi (82.9%), along with marine bacteria or marine actinomycetes (17.1%). The structural types of these reviewed compounds are mainly divided into polyketides (51.2%), terpenoids (21.0%), alkaloids (18.7%), amides or peptides (4.8%) and steroids (4.3%), while 8.5% of them are halogenated compounds.

## 2. Marine Microbial Anti-Inflammatory Compounds

### 2.1. Polyketides

In total, 129 polyketides with anti-inflammatory activity were obtained from marine microorganisms during 2021–2023.

**Figure 1:**
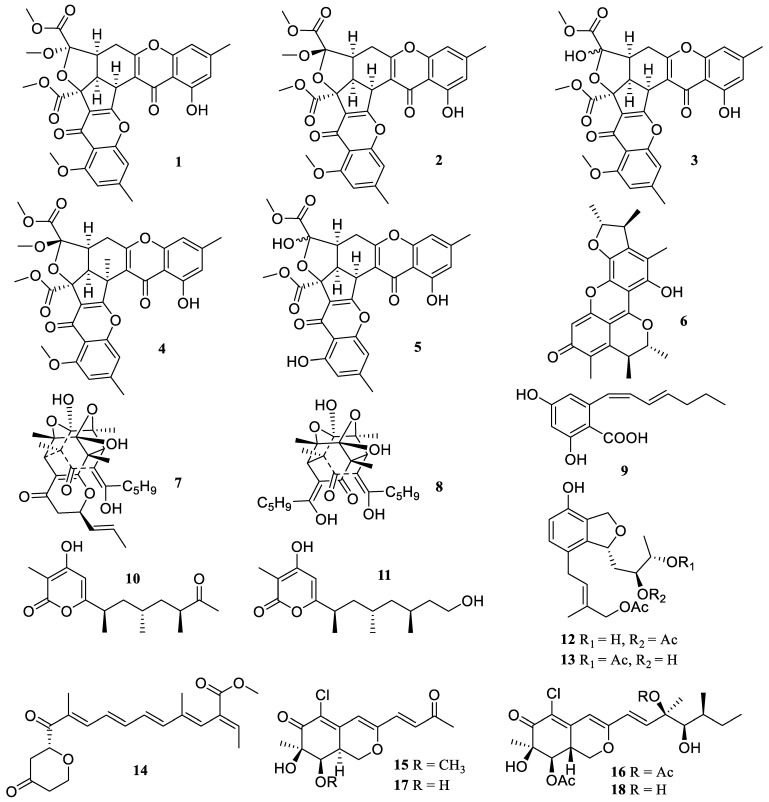
Chemical structures of polyketides (**1**–**18**).

Four rare chromone derivatives, epiremisporines D (**1**), E (**2**), G (**3**), and H (**4**), were isolated from marine-derived *Penicillium citrinum*, together with two known compounds, epiremisporine B (**5**) and penicitrinone A (**6**) (Figure 1). They significantly decreased *N*-Formyl-Met-Leu-Phe (fMLP)-induced superoxide anion generation by human neutrophils, with IC_50_ values of 6.4 ± 0.4, 8.3 ± 0.3, 31.7 ± 2.5, 33.5 ± 0.4, 3.6 ± 0.6, and 2.7 ± 0.1 µM, respectively [24,25].

Two known compounds, epitetrahydrotrichodimer ether (**7**) and tetrahydrotrichodimerol (**8**), were isolated and identified from the rhizosphere soil of *Hibiscus tiliaceus* Linn.-derived fungus *Penicillium* sp. DM 815. They inhibited the Gram-negative bacteria lipopolysaccharide (LPS)-induced upregulation of the inducible nitric oxide (NO) synthase (iNOS) at a concentration of 10 μM [26].

A new polyketide, 4-carboxy-5-((1*Z*,3*E*)-1,3-heptadien1-yl)-1,3-benzenediol (**9**), was obtained from the hydrothermal vent-derived fungus *Penicillium* sp. TW58-16. It markedly reduced the amount of NO released in RAW 264.7 cells upon exposure to LPS, which was consistent with a decrease in the production of inducible NO synthase (iNOS) at a concentration of 20 μM [27].

The chemical investigation of the fungus *Fusarium decemcellulare* SYSU-MS 6716 derived from a solid medium yielded two new polypropionate derivatives, decempyrones C (**10**) and J (**11**). Both demonstrated strong anti-inflammatory efficacy with IC_50_ values of 22.4 ± 1.8 and 21.7 ± 1.1 µM, respectively, by preventing LPS-induced NO generation in RAW 264.7 cells. Primary structure-activity relationships (SAR) analysis revealed that the alkyl side chain and pyrone functional groups are mainly responsible for the anti-inflammatory properties [28].

Two new compounds, heterocornols T (**12**) and X (**13**), were produced by the sponge-derived fungus *Pestalotiopsis heterocornis* XWS03F09 based on the one strain many compounds (OSMAC) approach. Both could reduce the amount of NO produced in response to LPS, which further significantly and dose-dependently reduced the expression of the iNOS protein in LPS-induced RAW 264.7 cells with 33 µM [29].

A chemical investigation of the seawater-derived fungus *Fusarium solani* 7227 yielded one new fusarin derivative, fusarin K (**14**). It exhibited strong anti-inflammatory activity (IC_50_ = 21.9 ± 9.8 µM) by preventing the generation of NO in RAW 264.7 cells that had been stimulated by LPS. The preliminary SAR study showed that the substituent group in polyunsaturated chain is primarily responsible for the anti-inflammatory properties [30].

The chemical study of the sponge-derived fungus *Penicillium sclerotiorum* E23Y-1A resulted in the isolation of two new azaphilones, penicilazaphilones F (**15**) and G (**16**), as well as two known analogs, hypocrellone A (**17**) and penicillazaphilone D (**18**). They reduced the LPS-induced NO generation in BV2 cells with IC_50_ values of 31.7 ± 1.5, 34.5 ± 1.4, 25.3 ± 2.2, and 34.8 ± 1.9 µM, respectively [31].

**Figure 2:**
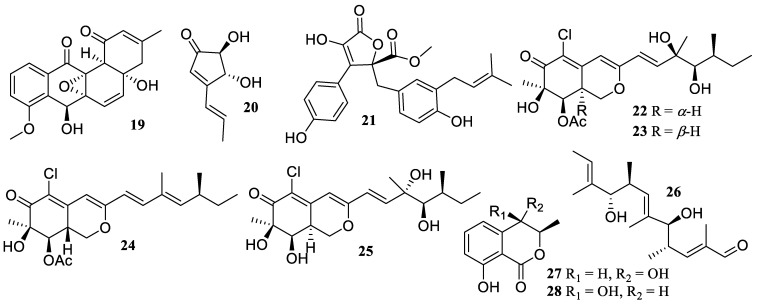
Chemical structures of polyketides (**19**–**28**).

One new compound, saccharothrixin G (**19**) (Figure 2), was obtained from the deep-sea sediment-derived fungus *Saccharothrix* sp. D09, which revealed inhibition on the production of NO with an IC_50_ value of 28 µM [32].

Two known metabolites, (+)-terrein (**20**) and butyrolactone I (**21**), were isolated and identified from a mangrove plant *Acanthus ilicifolius*-derived fungus, *Aspergillus flavipes* (MTCC 5220), which was collected from Goa, India. Both presented inhibitory activities of interleukine-6 (IL-6) and tumor necrosis factor-*α* (TNF-*α*) with IC_50_ values of 8.5 ± 0.7, 15.8 ± 0.2, 12.0 ± 0.9, and 43.3 ± 0.8 µM, respectively, whereas **21** demonstrated low toxicity to host cells in LPS-stimulated THP-1 cells [33]. Moreover, compound **21** showed noteworthy activity by blocking the release of neutrophil elastase with an IC_50_ value of 2.3 ± 0.3 µM, which was isolated from the annelid *Spirorbis* sp.-derived fungus *Aspergillus terreus* MT 273950 [34].

The chemical study of the alga-derived fungus *Penicillium sclerotiorum* Al-27 yielded one new azaphilone, 8a-*epi*-hypocrellone A (**22**), as well as two known azaphilones, hypocrellone A (**23**) and isochromophilone IV (**24**). They inhibited the TNF-*α*-induced nuclear factor-*κ*B (NF-*κ*B) phosphorylation but without changing the NF-*κ*B activity at a concentration of 20 µM [35]. Two known azaphilone derivatives, compounds **24** and WB (**25**), were produced by co-culturing the mangrove endophytic fungus *P. sclerotiorum* THSH–4 with *P. sclerotiorum* ZJHJJ-18 in PDB medium. When compared to the positive control, indomethacin (IC_50_ = 35.3 µM), both showed a stronger suppression of LPS-induced NO release from RAW 264.7 with IC_50_ values of 17.6 and 4.7 µM, respectively, without clearly deleterious effects within 50 µM [36].

Three known metabolites, 5,9-dihydroxy-2,4,6,8,10-pentamethyldodeca-2,6,10-trienal (**26**), (3*R*, 4*S*)-(−)-4-hydroxymellein (**27**), and (3*R*, 4*R*)-(−)-4-hydroxymellein (**28**), were isolated from the alga *Hypnea pannosa*-derived fungus *Aspergillus ochraceopetaliformis* SCSIO 41020. They illustrated a dose-dependent inhibitory effect against the excessive generation of NO and pro-inflammatory cytokines in LPS-treated RAW 264.7 macrophages without cytotoxicity at a concentration of 10 µM. Moreover, compound **28** inhibited the release of pro-inflammatory cytokines (IL-6, MCP-1, and TNF-*α*) when LPS was applied in both in vitro and in vivo settings [37].

**Figure 3:**
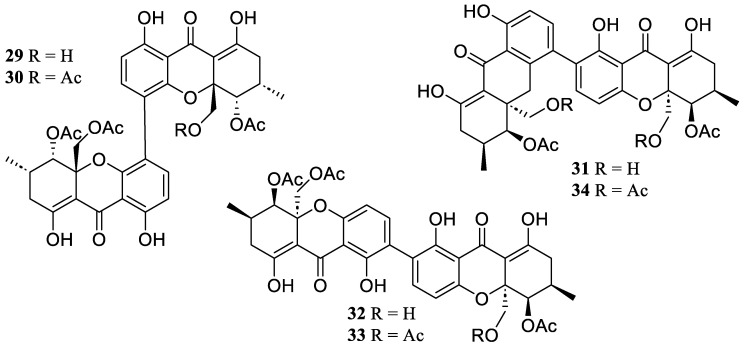
Chemical structures of polyketides (**29**–**34**).

Six known xanthone dimeric analogs were obtained from the ascidian *Styela plicata*-derived fungus *Diaporthe* sp. SYSU-MS 4722, which were 12-deacetylphomoxanthone A (**29**), phomoxanthones A (**30**) and B (**31**), dicerandrols B (**32**) and C (**33**), and deacetylphomoxanthone B (**34**) (Figure 3). They indicated anti-inflammatory activity with IC_50_ values ranging from 6.3 to 8.0 µM, which suppressed toward NO generation in LPS-induced RAW 264.7 [38].

**Figure 4:**
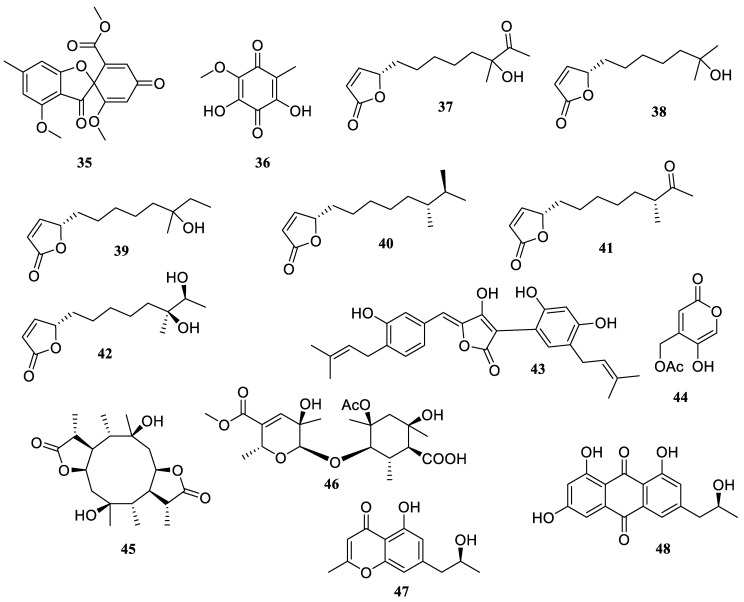
Chemical structures of polyketides (**35**–**48**).

The fungus *Talaromyces helicus* SCSIO 41311, which is derived from cold seeps in the South China Sea, was shown to contain two distinct compounds, trypacidin (**35**) and fumiquinone B (**36**) (Figure 4). They displayed NO inhibitions with IC_50_ values of 38.6 and 15.5 µM, respectively. Interestingly, compound **36** showed a greater inhibitory effect of NO compared to the positive control, eicosapentaenoic acid (IC_50_ = 50.0 μM) [39].

The chemical investigation of marine sediment-derived actinomyces *Streptomyces* sp. 13G036 yielded six known butenolides, (4*S*)-4,10-dihydroxy-10-methyl-11-oxo-dodec-2-en-1,4-olide (**37**), (4*S*)-4,10-dihydroxy-10-methyl-undec-2-en-1,4-olide (**38**), (4*S*)-4,10-dihydroxy-10-methyl-dodec-2-en-1, 4-olide (**39**), (4*S*,10*R*,11*S*)-4,11-dihydroxy-10-methyl-dodec-2-en-1,4-olide (**40**), (4*S*)-4-hydroxy-10-methyl-11-oxo-dodec-2-en-1,4-olide (**41**), and (4*S*,10*S*,11*S*)-4,10,11-trihydroxy-10-methyl-dodec-2-en-1,4-olide (**42**). They showed anti-inflammatory properties by preventing the generation of NO, TNF-*α*, and IL-6 in LPS-stimulated macrophages at a concentration of 10 µM [40].

One new compound, aspulvinone V (**43**), together with two known compounds, (+)-terrein (**20**) and butyrolactone I (**21**), were isolated and identified from a marine green alga *Ulva lactuca* L.-derived fungus, *Aspergillus terreus* Thom (Trichocomaceae) strain NTU 243, that was collected from Taiwan’s northeast coast. By quantifying the quantity of NO generation in LPS-induced BV2 cells, all isolates were evaluated for their anti-inflammatory action. At a dosage of 10 µM, the isolates showed inhibition rates of 45.0%, 49.2%, and 34.5%, respectively [41].

Saadamysin (**44**) was characterized from the coral-associated *Aspergillus flavus* GXIMD 02503, which demonstrated moderate inhibitory actions of NF-*κ*B activation with an IC_50_ value of 10.7 ± 1.3 µM [42]. The chemical investigation of the sponge-derived fungus *Pestalotiopsis* sp. SWMU-WZ04-2 yielded two new compounds, pestaloketides A (**45**) and B (**46**). Both reduced the activity of NO generation produced by LPS with IC_50_ values of 23.6 and 14.5 µM, respectively, without observed cytotoxicity [43].

Two known compounds, isorhodoptilometrin (**47**) and 5-hydroxy-7-(2′-hydroxypropyl)-2-methyl-chromone (**48**), were discovered from the sponge-derived fungus *Penicillium oxalicum* CLC-MF 05. These compounds inhibited the overproduction of NO and prostaglandin E2 (PGE_2_), as well as the overexpression of iNOS and cyclooxygenase-2 (COX-2) in both LPS-stimulated BV2 and rat primary microglia [44].

**Figure 5:**
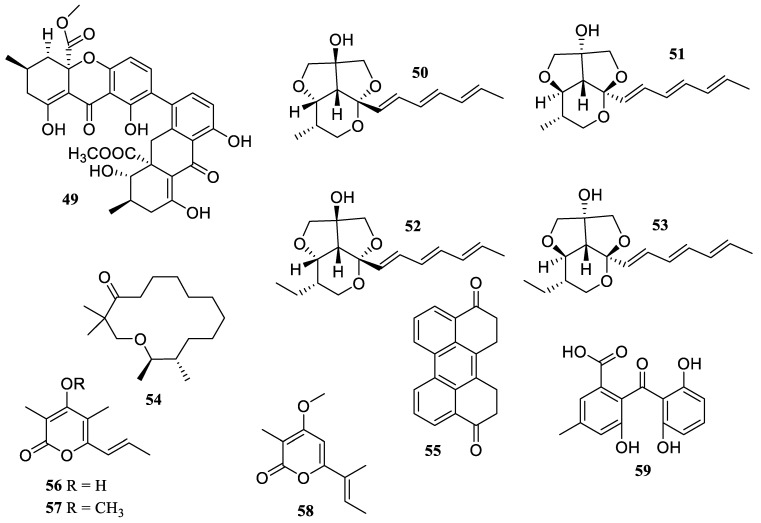
Chemical structures of polyketides (**49**–**59**).

The soft coral-associated fungus *Aspergillus* sp. SCSIO 41036 was the source of one known compound, penicillixanthone A (**49**) (Figure 5). It exhibited an inhibitory effect against NO induced by LPS in RAW 264.7 cells at a dosage of 10 µM [45]. The chemical investigation of *Stratomyces specialis* 208DD-067, an actinomycete obtained from sediment, yielded four new streptoglycerides E–H (**50**–**53**) with a unique 6/5/5/-membered ring structure. They demonstrated strong anti-inflammatory efficacy with IC_50_ values of 10.9, 5.9, 4.7, and 3.5 µM, respectively, in suppressing LPS-induced NO generation in RAW 264.7 cells [46].

A chemical investigation of the mangrove endophytic fungus *Daldinia eschscholtzii* KBJYZ-1 yielded two new polyketides, eschscholin B (**54**) and daldilene A (**55**). They exhibited noteworthy anti-inflammatory properties, with IC_50_ values of 19.3 and 12.9 µM, respectively. Furthermore, compound **54** reduced the expression of COX-2 and iNOS in RAW 264.7 cells that had been exposed to LPS. Further molecular biology study revealed the potential mechanism of compound **54**’s anti-inflammatory function by inactivating the MAPK and NF-*κ*B signaling pathways [47].

The chemical investigation of marine ascidian-derived fungus *Amphichorda felina* SYSU-MS 7908 resulted in the isolation of two new *α*-pyrone derivatives, amphichopyrones A (**56**) and B (**57**). Both displayed potent anti-inflammatory activity by inhibiting the production of NO in RAW 264.7 cells with IC_50_ values 18.1 ± 4.8 and 7.2 ± 0.9 µM, respectively [48].

Two known polyketides, nectriapyrone (**58**) and monodictyphenone (**59**), were also obtained from marine ascidian-derived *Diaporthe* sp. SYSU-MS 4722. Both indicated anti-inflammatory efficacy by preventing LPS-induced NO production with IC_50_ values of 35.4 and 40.8 µM, respectively (IC_50_ = 35.8 µM for the positive control, indomethacin) [49].

**Figure 6:**
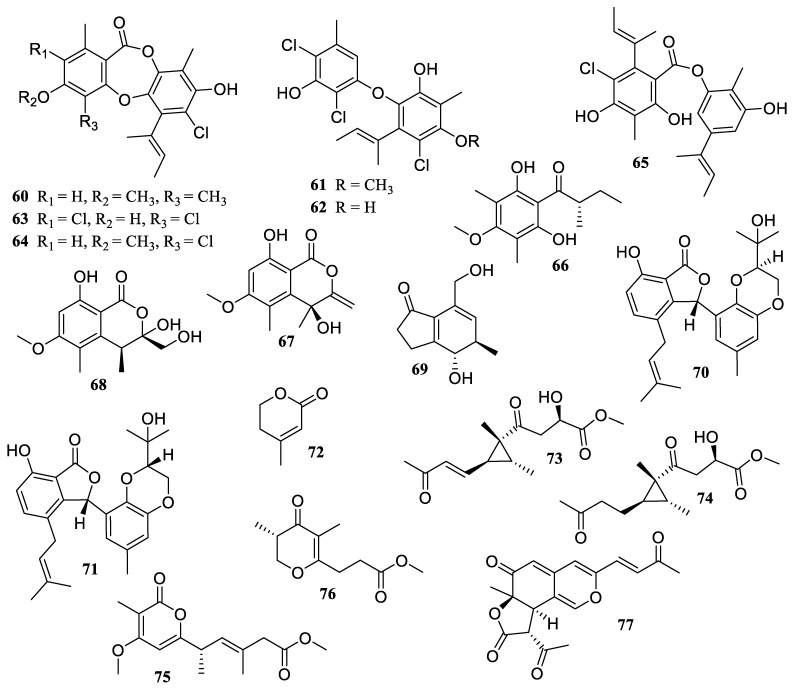
Chemical structures of polyketides (**60**–**77**).

The chemical investigation of the Beibu Gulf coral-derived fungus *Aspergillus unguis* GXIMD 02505 yielded a new depsidone derivative, aspergillusidone H (**60**), and six known biosynthetically related chlorinated polyketides: aspergillus ethers J (**61**) and F (**62**), nornidulin (**63**), aspergillusidone B (**64**), guisinol (**65**), and 1-(2,6-dihydroxy-4-methoxy-3,5-dimethylphenyl)-2-methylbutan-1-one (**66**) (Figure 6). They demonstrated suppression of LPS-induced NF-*κ*B in RAW 264.7 macrophages at a concentration of 20 µM. Furthermore, the two potent inhibitors (**62** and **65**) dose-dependently reduced the receptor activator of NF-*κ*B ligand (RANKL)-induced osteoclast differentiation in bone marrow macrophage cells (BMMs) without obvious cytotoxicity [50].

The chemical examination of the marine-derived fungal species *Eutypella scoparia* yielded two known compounds, 4,8-dihydroxy-6-methoxy-4,5-dimethyl-3-methyleneisochroman-1-one (**67**) and banksialactone A (**68**). Both illustrated anti-inflammatory properties by inhibiting LPS-induced NO generation in RAW 264.7 macrophages, with inhibition rates of 49.0% and 54.9% at 50.0 µg/mL, respectively [51].

A new indanone derivative, streptinone (**69**), was isolated and identified from a marine sediment-derived *Streptomyces massiliensis* 213DD-128, which suppressed the production of NO, PGE_2_, and pro-inflammatory cytokines, such as TNF-*α*, IL-6, and interleukin-1 beta (IL-1*β*), by inhibiting the TLR-mediated NF-*κ*B signaling pathway at a concentration of over 5 µM [52].

Secondary metabolites of a deep-sea sediment sample-derived fungus, *Phomopsis lithocarpu*s FS 508, were investigated, including three known compounds, lithocarol F (**70**), isoprenylisobenzo-furan A (**71**), and anhydromevalonolactone (**72**). They showed significant anti-inflammatory activities on LPS-induced NO production in RAW 264.7 macrophages, with IC_50_ values of 22.8, 27.2, and 24.1 µM, respectively, all of which were superior to the positive control, indometacin (IC_50_ = 32.9 µM) [53].

The chemical investigation of *Stragonospora* sp. SYSU-MS 7888, a fungus originating from sponges in the South China Sea, provided two new cyclopropane derivatives, stagonospones A (**73**) and B (**74**), and two new *α*-pyrone derivatives, stapyrones E (**75**) and G (**76**). They displayed considerable anti-inflammatory efficacy by suppressing LPS-induced NO generation with IC_50_ values of 3.6 ± 1.0, 9.4 ± 1.8, 21.9 ± 3.5, and 22.8 ± 3.9 µM, respectively, surpassing that of the positive control, indomethacin (IC_50_ = 26.5 ± 1.1 µM). The double bond at C-3 in the family of cyclopropane diones may increase cytotoxicity and thereby boost anti-inflammatory efficacy. Meanwhile, the anti-inflammatory properties of pyrones were dependent on the side chain length and ketone position [54].

One new azaphilone, penicilazaphilone N (**77**), was produced by the sponge-derived fungus *Penicillium sclerotiorum* E23Y-1A. It presented moderate anti-inflammatory efficacy by preventing LPS-induced NO production with an IC_50_ value of 22.6 ± 3.0 µM [55].

**Figure 7:**
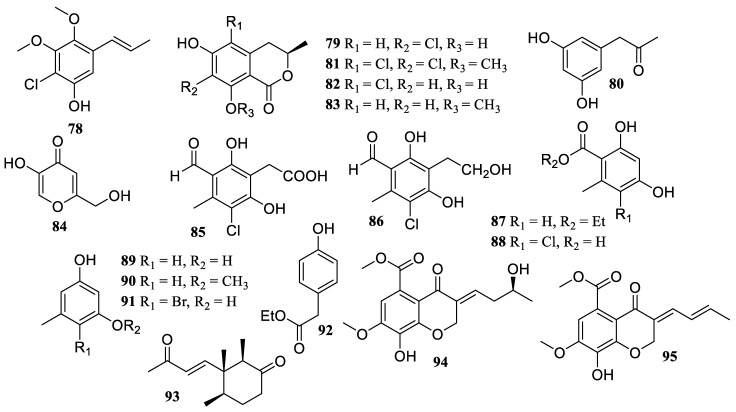
Chemical structures of polyketides (**78**–**95**).

One new propenylphenol derivate, chlomophenol A (**78**), together with six known compounds, 7-chloro-3,4-dihydro-6,8-dihydroxy-3-methylisocoumarine (**79**), *α*-acetylorcinol (**80**), (*S*)-5,7-dichloro-6-methoxy-2-methyl-2,3-dihydrobenzofuran-4-carboxylic acid (**81**), 5-chloro-6-hydroxymellein (**82**), 3-methyl-6-hydroxy-8-methoxy-3,4-dihydroisocoumarin (**83**), and kojic acid (**84**) (Figure 7), were obtained from a mangrove-endophytic fungus *Amorosia* sp. SCSIO 41026. They showed inhibitory effects on the overproduction of NO and pro-inflammatory cytokines in LPS-induced RAW 264.7 macrophages without cytotoxicity at a concentration of 10 µM [56].

Two new chlorinated orsellinic aldehyde derivatives, orsaldechlorins A (**85**) and B (**86**), as well as seven known analogs, ethyl orsellinate (**87**), 5-chloroorsellinic acid (**88**), orcinol (**89**), *O*-methylorcinol (**90**), aryl bromide (**91**), ethyl 4-hydroxyphenylacetate (**92**), and nectriatone C (**93**), were identified from the Beibu Gulf coral-derived fungus *Acremonium sclerotigenum* GXIMD 02501. They displayed suppression of NF-*κ*B activation triggered by LPS in RAW 264.7 cells at the dosage of 20 µM. Additionally, the two new potent inhibitors (**85** and **86**) inhibited RANKL-induced osteoclast differentiation in BMMs without cytotoxicity [57].

The chemical investigation of the mangrove-derived fungus *Diaporthe* sp. XW12–1 resulted in the isolation of two new chromone compounds, diaporspchromanones B (**94**) and C (**95**). Both demonstrated anti-inflammatory activity by inhibiting LPS-induced NO production with IC_50_ values of 19.1 ± 3.6 and 9.6 ± 0.2 µM, respectively, which were stronger than that of the positive control, indomethacin (IC_50_ = 70.3 ± 1.0 µM) [58].

**Figure 8:**
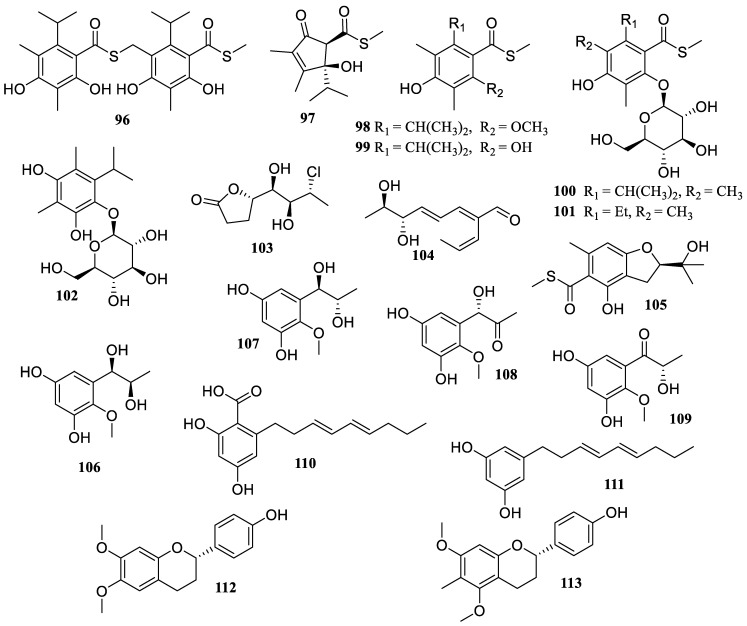
Chemical structures of polyketides (**96**–**113**).

The fungus *Streptomyces* sp. DS-27 was originated from the rhizosphere of marine cordgrass *Spartina alterniflora*. The chemical investigation of its cultures produced two new compounds, streptothiomycin E (**96**) and *S*-methyl (4*R*,5*S*)-2,3-dimethyl-4-hydroxy-4-isopropyl-1-oxocyclopent-3-ene-5-carbothioate (**97**) (Figure 8). Both showed potential anti-inflammatory effects by reducing NO concentration levels in a dose-dependent manner (ranging from 2.5 to 40 µM) [59].

The sediment-derived *Streptomyces* sp. ZSN 77 was found to contain four new compounds, suncheonosides E (**98**), F (**99**), J (**100**), and *S*-methyl 4-hydroxy-6-isopropyl-2-methoxy-3,5-dimethylbenzothioate (**101**), along with one known compound, *S*-methyl 2,4-dihydroxy-6-isopropyl-3,5-dimethylbenzothioate (**102**). They exhibited in vivo anti-inflammatory activity through the suppression of NO generation. Compound pretreatment resulted in a dose-dependent (ranging from 2.5 to 10 µM) significant reduction in the concentration of NO [60].

*Neofusicoccum parvum* Y2NBKZG 1016, a fungus derived from the fruits of mangrove plant *Sonneratia glauca*, produced a new compound, (4*S*,5*S*,6*S*,7*R*)-4-(3-chloro-1,2-dihydroxybutyl)-butyrolactone (**103**). It presented a minimal anti-inflammatory effect at doses ≥6.3 µM, attaining a maximum inhibition rate of 28.9% without causing cytotoxicity against RAW 264.7 cells [61].

The chemical examination of the seaweed *Caulerpa* sp.-derived fungus *Talaromyces cyanescens* yielded one new compound, talacyanol B (**104**), and one known polyene molecule, eurothiocin A (**105**). Both reduced the generation of NO and the expression of COX-2 and iNOS in BV2 cells that were triggered by LPS at concentrations of 50, 100, and 200 µM, respectively [62].

Four new phenolic compounds, asperpropanols A–D (**106**–**109**), and two known congeners, 2,4-dihydroxy-6-((3*E*,5*E*)-nona-3,5-dien-1-yl)-benzoic acid (**110**) and 5-[(3*E*,5*E*)-3,5-nonadienyl]-1,3-benzenediol (**111**), were discovered from the deep-sea sediment-derived fungus *Aspergillus puniceus* SRRC 2155. They showed anti-inflammatory effect on LPS-induced RAW 264.7 cells by reducing the generation of NO, TNF-*α*, and IL-6 at a dosage of 20 µM [63].

The chemical investigation of the mangrove soil-derived *Isoptericola chiayiensis* BCRC 16888 yielded two new flavonoids, chiayiflavans D (**112**) and E (**113**). Both exhibited stronger NO inhibitory activity than that of the positive control, quercetin (IC_50_ = 37.0 µM), with IC_50_ values of 17.1 and 9.4 µM, respectively [64].

**Figure 9:**
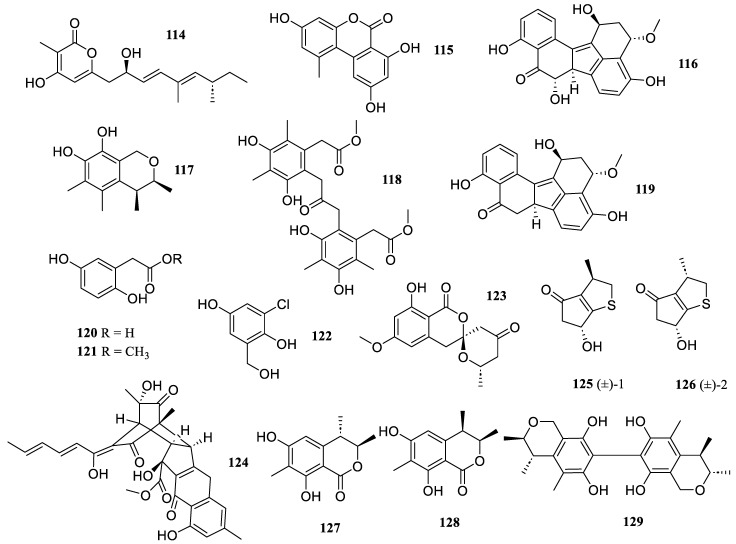
Chemical structures of polyketides (**114**–**129**).

One new *α*-pyrone derivatives, diaporpyrone A (**114**), was isolated from cultures of the mangrove endophytic fungus *Diaporthe* sp. QYM 12 (Figure 9). It inhibited the production of NO in LPS-induced RAW 264.7 cells with an IC_50_ value of 12.5 µM [65]. The chemical examination of the Antarctic fungi *Pleosporales* sp. SF-7343 revealed one known fungal metabolite, alternariol (**115**). It inhibited the secretion of interleukin-8 and -6 in TNF-*α*/interferon-*γ*-treated HaCaT cells at concentrations of 2.5 to 10.0 µM [66].

Three new compounds, guhypoxylonols A (**116**), C (**117**), and D (**118**), were isolated from the mangrove endophytic fungus *Aspergillus* sp. GXNU-Y45, together with one previously reported metabolite, hypoxylonol B (**119**). They presented inhibitory activity against the production of NO, with IC_50_ values of 14.4 ± 0.1, 18.0 ± 0.1, 16.7 ± 0.2, and 21.1 ± 0.1 µM, respectively [67].

The chemical investigation of the marine sponge-derived fungal strain *Aspergillus* sp. IMBC-FP2.05 resulted in the isolation of three compounds, namely, homogentisic acid (**120**), methyl (2,5-dihydroxyphenyl) acetate (**121**), and 3-chloro-2,5-dihydroxybenzyl alcohol (**122**). They demonstrated the most inhibitory effects against NO overproduction, with IC_50_ values of 28.2, 14.2, and 41.8 µM, respectively, which was comparable with that of the positive control, N^G^-Monomethyl-L-arginine (L-NMMA) (IC_50_ = 44.5 µM) [68].

One new unique isocoumarin, penicillol B (**123**), was isolated from the mangrove endophytic fungus *Penicillium* sp. BJR-P2. It inhibited LPS-induced NO production in RAW 264.7 cells, with an IC_50_ value of 12.0 µM, which was more potent than that of the positive control, indomethacin (IC_50_ = 35.8 ± 5.7 µM). A docking study revealed that it was perfectly docking into the active site of murine inducible NO oxygenase (iNOS) by forming multiple typical hydrogen bonds [69].

Guided by MS/MS-based molecular networking, bisorbicillchaetone B (**124**), a new hybrid sorbicillinoid, was isolated from cultures of the sediment-derived fungus *Penicillium* sp. SCSIO 06868. It exhibited inhibitory effect on NO production in LPS-activated RAW 264.7 cells with an IC_50_ value of 38.4 ± 3.3 μM, without cytotoxicity observed [70].

Ochrathinols A (**125**) and B (**126**), two new sulfur-containing racemates, were isolated from an Antarctic soil-derived fungus, *Aspergillus ochraceopetaliformis* SCSIO 05702. They were obtained as unprecedented sulfur natural products featuring a novel 3-methylhexahydro-2H-cyclopenta [*b*]thiophene core, which suppressed the release of LPS-induced IL-1*β*, IL-6, and TNF-*α* inflammatory cytokines at a concentration of 10.0 μM and alleviated the unbalanced NAD^+^/NADH ratio caused by LPS in RAW 264.7 macrophages [71].

Three known compounds, (3R*,4S*)-6,8-dihydroxy-3,4,7-trimethylisocoumarin (**127**), sclerotinin C (**128**), and asperbiphenyl (**129**), were isolated from the sediment-derived *Penicillium citrinum* W 17. They exhibited significant inhibitory effects on LPS-stimulated NO production in murine brain microglial BV2 cells in a dose-dependent manner under concentrations of 2.5, 5.0, and 10.0 µM, respectively [72].

### 2.2. Terpenoids

In total, 53 terpenoids with anti-inflammatory activity were obtained from marine microorganisms during 2021–2023, comprising 29 sesquiterpenes, 4 diterpene, 15 triterpenoids, and 5 meroterpenoids.

#### 2.2.1. Sesquiterpenes

**Figure 10:**
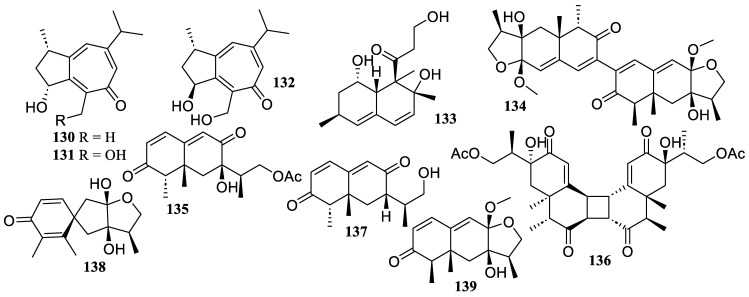
Chemical structures of sesquiterpenoids (**130**–**139**).

The chemical investigation of the deep-sea sediment-derived fungus *Spiromastix* sp. MCCC 3A00308 yielded three new sesquiterpenes, spiromaterpenes D–F (**130**–**132**) (Figure 10). The NO production on LPS-induced microglia cells BV2 was significantly inhibited by them, with IC_50_ values of 26 ± 2, 9 ± 1, and 20 ± 1 µM, respectively. The preliminary SAR analyses demonstrated that compound **131** with a 2,11-diol significantly increased the inhibitory effect [73].

A known sesquiterpene, decumbenone A (**133**), was obtained from the Indian Ocean 30 m deep water-derived fungus *Aspergillus austroafricanus* Y32-2, which was found to exhibit a dose-dependent anti-inflammatory activity at concentrations of 30 to 120 µg/mL, by using a zebrafish inflammation model caused by copper sulfate [74].

Five new sesquiterpenes, paraconulones B–E (**134**–**137**) and G (**138**), along with a known sesquiterpene, 4-*epi*-microsphaeropsisin (**139**), were isolated and identified from coastal sediment-derived from *Paraconiothyrium sporulosum* DL-16. They showed inhibitory effects on LPS-induced NO production in BV2 cells with IC_50_ values of 6.9 ± 2.6, 7.7 ± 2.0, 2.8 ± 0.5, 8.1 ± 2.9, 8.1 ± 3.5, and 4.6 ± 3.5 µM, respectively, which were comparable to the positive control, curcumin (IC_50_ = 8.6 ± 1.6 µM) [75].

**Figure 11:**
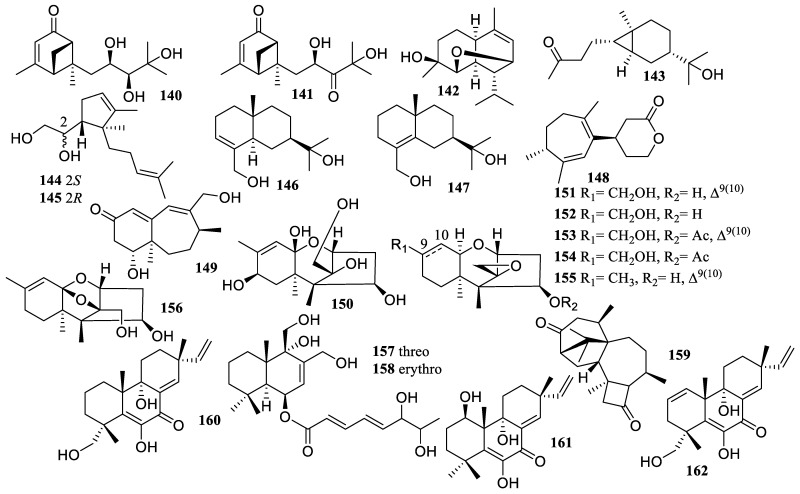
Chemical structures of sesquiterpenoids and diterpene (**140**–**162**).

The chemical examination of the deep-sea sediment-derived fungus *Eutypella* sp. MCCC 3A00281 resulted in the isolation of eight sesquiterpenes, including six new ones, eutypeterpenes B (**140**) and C (**141**), eutypeterpene M (**142**), eutypeterpene N (**143**), and eutypeterpenes P (**144**) and Q (**145**), and two known ones, eudesma-3-en-11,15-diol (**146**) and eudesma-4-en-11,15-diol (**147**) (Figure 11). They illustrated inhibitory effects on LPS-induced NO production in RAW 264.7 macrophages with IC_50_ values of 13.4 ± 0.8, 16.8 ± 1.0, 11.8 ± 1.0, 8.6 ± 1.0, 14.3 ± 1.1, 11.5 ± 1.2, 18.3 ± 1.0, and 17.1 ± 1.0 µM, respectively. In addition, compounds **140**–**145** demonstrated stronger activity than that of quercetin (IC_50_ = 17 ± 1.5 µM) [76].

Two new sesquiterpenoids, nigerin (**148**) and ochracene J (**149**), were obtained from the South China Sea sponge *Dysidea* sp. symbiotic fungus *Aspergillus niger*. Both exhibited strong inhibitory effects on the generation of NO in LPS-stimulated RAW 264.7 macrophages with IC_50_ values of 8.5 and 4.6 µM, respectively [77].

Seven trichothecenes, including three new compounds, (2*R*,4*R*,5*S*,5a*R*,7*R*,9a*S*,10*S*)-10-(hydroxymethyl)-5,5a,8-trimethyl-3,4,5,5a,6,7-hexahydro-2,5-methanobenzo[*b*]oxepine-4,7,9a,10(2*H*)-tetraol (**150**), (2*S*,2′*R*,4′*R*,5′*S*,5a′*R*,9a′*R*)-8′-(hydroxymethyl)-5′,5a′-dimethyl-2′,3′,4′,5′,5a′,6′,7′,9a′-octahydrospiro[oxirane-2,10′-[2,5] methanobenzo[*b*]oxepin]-4′-ol (**151**), and (2*S*,2′*R*,4′*R*,5′*S*,5a′*R*,9a′*R*)-8′-(hydroxymethyl)-5′,5a′-dimethyldecahydrospiro[oxirane-2,10′-[2,5]methanobenzo[*b*]oxepin]-4′-ol (**152**), and four known ones, trichoderminol (**153**), trichodermarins A (**154**) and E (**155**), and trichodermol (**156**), were isolated from marine alga *Mastophora rosea*-derived fungus *Trichoderma brevicompactum* NTU 439. Compounds **150**–**154** and **156** displayed minimal inhibitory effects against BV2 cells without cytotoxicity at a dosage of 10 µM. Additionally, compound **159** showed a substantial inhibitory effect on the generation of NO caused by LPS with an IC_50_ value of 5.2 ± 0.4 µM [78].

Meanwhile, two new drimane sesquiterpenes, ustusolates H (**157**) and I (**158**), were isolated from a seagrass-derived fungus, *Aspergillus insuetus* SYSU 6925. Both exhibited a potent inhibition of NO production in RAW 264.7 cells with IC_50_ values of 21.5 ± 1.1, and 32.6 ± 1.2 µM, respectively [79].

#### 2.2.2. Diterpene

A known compound, hazianol J (**159**), was obtained from the deep-sea sediment-derived fungus *Trichoderma* sp. SCSIOW 21, which showed anti-inflammatory activity at 100 µM with a NO inhibition rate of 81.8% [80].

The chemical examination of the fermentation broth of *Eutypella* sp. D-1, using the OSMAC strategy of adding ethanol as a promoter in the culture medium, resulted in the isolation of one new compound, libertellenone Z (**160**), and two known compounds, libertellenones A (**161**) and C (**162**). They exhibited strong NO inhibition rates of 60.9%, 89.4%, and 84.2% at 10.0 µM, respectively, while the latter two were superior to the effect of the positive drug dexamethasone with rates of 72.0% at 10.0 µM [81].

#### 2.2.3. Triterpenoids

**Figure 12:**
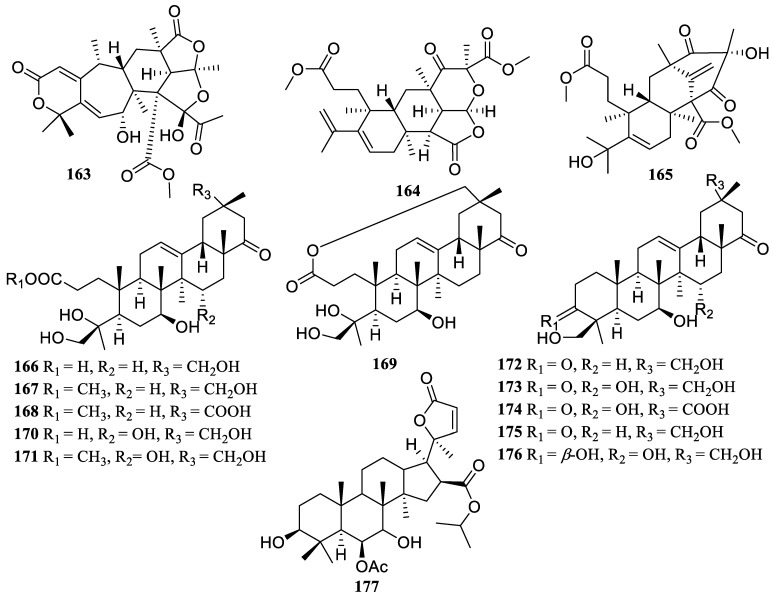
Chemical structures of triterpenoids (**163**–**177**).

Three new compounds, peniscmeroterpenoids A (**163**), D (**164**), and L (**165**), were isolated from the marine-derived fungus *Penicillium sclerotiorum* GZU-XW03-2 (Figure 12), which inhibited the production of NO in RAW 264.7 cells with IC_50_ values of 26.6 ± 1.2, 8.8 ± 1.2, and 48.0 ± 2.5 µM, respectively. Moreover, compound **164** further significantly suppressed the production of pro-inflammatory mediators, tumor necrosis COX-2, IL-1*β*, and IL-6 and the protein expression of the enzyme iNOS [82,83].

Moreover, soyasapogenols B1–B11(**166**–**176**) were identified from marine actinomycete *Nonomuraea* sp. MYH 522. These compounds presented anti-inflammatory effects in DMXAA-stimulated RAW 264.7 cells by suppressing the STING/TBK1/NF-*κ*B pathway at a concentration of 20 µM [84].

The chemical investigation of the alga-derived fungus *Turbinaria decurrens* yielded one new compound, decurrencyclic B (**177**). It showed superior attenuation properties against COX-2 and 5-lipoxygenase with IC_50_ values of 14.0 and 3.0 µM, respectively [85].

#### 2.2.4. Meroterpenoids

**Figure 13:**
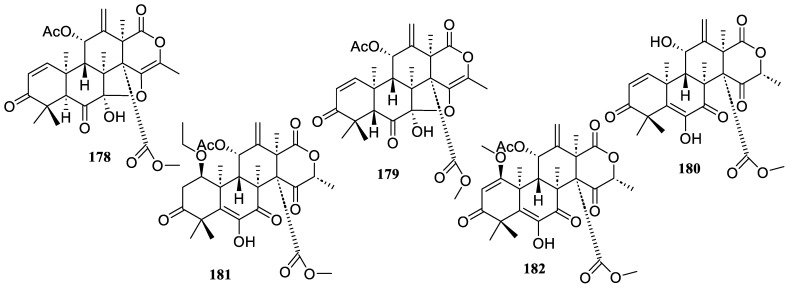
Chemical structures of meroterpenoids (**178**–**182**).

The chemical investigation of marine-derived fungus *Aspergillus terreus* GZU-31-1 yielded five new congeners, aspermeroterpenes D–H (**178**–**182**) (Figure 13). These compounds prevented RAW 264.7 cells from producing NO in response to LPS. They demonstrated notable anti-inflammatory activity with IC_50_ values of 6.7 ± 0.8, 29.6 ± 3.9, 22.2 ± 0.9, 25.9 ± 3.1, and 26.5 ± 1.0 µM, respectively [86].

### 2.3. Alkaloids

In total, 47 alkaloids with anti-inflammatory activity were obtained from marine microorganisms during 2021–2023.

**Figure 14:**
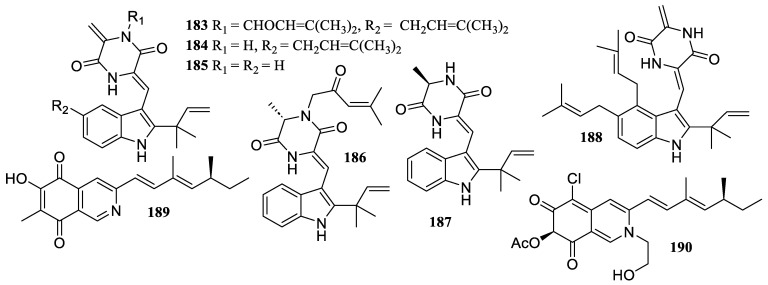
Chemical structures of alkaloids (**183**–**190**).

Two new compounds, aspechinulins B (**183**) and C (**184**), together with four known compounds, isoechinulins A (**185**) and B (**186**), neoechinulin B (**187**), and cryptoechinuline G (**188**) (Figure 14), were isolated from the sediment-derived fungus *Aspergillus* sp. FS 445. They illustrated inhibitory effects against NO production with IC_50_ values ranging from 20 to 90 µM [87].

The chemical examination of co-cultures of *Penicillium sclerotiorum* THSH–4 and *Penicillium sclerotiorum* ZJHJJ–18 produced one new azaphilone, peniazaphilone A (**189**), and one known azaphilone, isochromophilone VI (**190**). Both revealed a strong suppression of LPS-induced NO release from RAW 264.7 without cytotoxicity with IC_50_ values of 7.1 and 17.0 µM, respectively [36].

**Figure 15:**
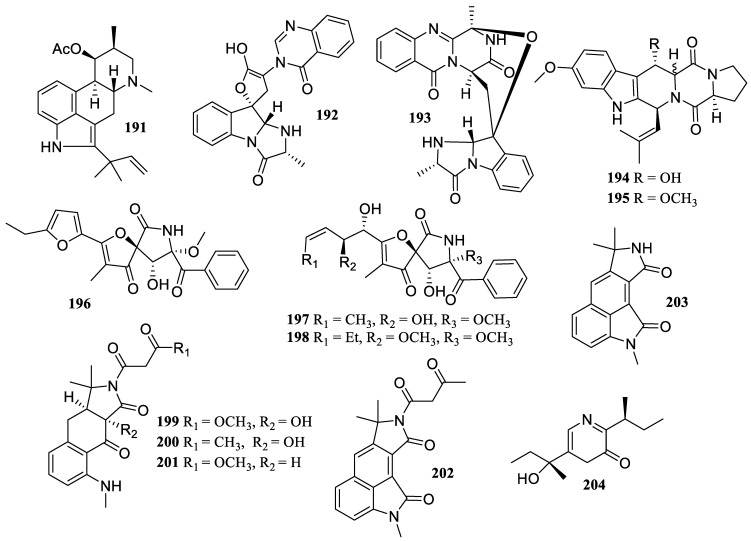
Chemical structures of alkaloids (**191**–**204**).

Eight known compounds, fumigaclavine C (**191**), isotryptoquivaline F (**192**), fumiquinazoline F (**193**), 12,13-dihydroxyfumitremorgin C (**194**), cyclotryprostatin B (**195**), azaspirofuran A (**196**), 14-norpseurotin A (**197**), and 11-*O*-methylpseurotin A (**198**) (Figure 15), were isolated from the fungus *Talaromyces helicus* SCSIO 41311. They showed moderate NO inhibitory activity with IC_50_ values of 23.5, 26.5, 21.4, 25.0, 29.6, 9.7, 32.4, and 32.2 µM, respectively [39].

In addition to a new oxygenated tricyclic cyclopiazonic acid, asperorydine Q (**199**), the chemical study of the fungus *Aspergillus flavus* GXIMD 02503 produced five known compounds, asperorydines O (**200**) and J (**201**), speradine H (**202**), cyclopiamide A (**203**), and pyrazinemethanol (**204**). They presented suppression of LPS-induced NF-*κ*B activation with IC_50_ values of 14.1 ± 1.5, 21.8 ± 1.9, 8.6 ± 1.3, 17.4 ± 1.7, 11.3 ± 2.0, and 6.5 ± 1.4 µM, respectively [42].

**Figure 16:**
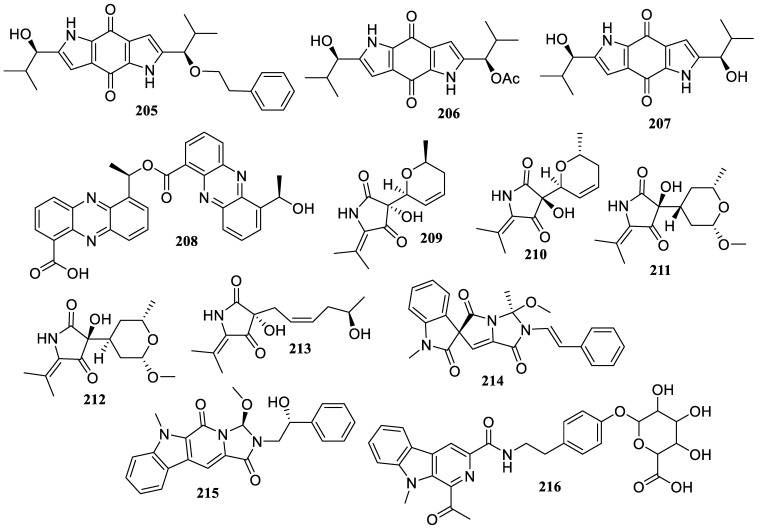
Chemical structures of alkaloids (**205**–**216**).

The chemical investigation of a sponge-derived fungus, *Aspergillus tamarii* MCCF 102, resulted in the isolation of two new dipyrrolobenzoquinones, terreusinones B (**205**) and C (**206**), along with one known analog, terreusinone (**207**) (Figure 16). They showed anti-inflammatory activity by inhibiting NO production in a dose-dependent manner (IC_50_ < 1 µM) without any cytotoxicity [88].

Furthermore, a strain of *Cystobasidium laryngis* obtained from deep-sea sediments of the Indian Ocean Ridge produced phenazostatin J (**208**), a new diphenazine derivative. It displayed significant anti-neuroinflammatory activity with an IC_50_ value of 0.3 μM, without cytotoxicity at a concentration of over 1.0 μM [89].

Five new compounds, lecanicilliumins A (**209**), B (**210**), E (**211**), F (**212**), and G (**213**), were obtained from the sediment-derived fungus *Lecanicillium fusisporum* GXIMD 00542. They demonstrated moderate anti-inflammatory activity by reducing LPS-induced NF-*κ*B activation in RAW 264.7 cells with inhibition rates of 50% at 18.5 ± 1.2, 25.8 ± 1.3, 23.1 ± 1.3, 24.7 ± 1.2, and 26.5 ± 1.1 µM, respectively [90].

The chemical examination of marine sponge *Phakellia fusca*-associated fungus *Actinoalloteichus cyanogriseus* LHW 52806 produced one new *β*-carboline compound, marinacarboline glucuronide (**214**), as well as two known compounds, marinacarboline L (**215**) and cyanogramide (**216**). They showed anti-inflammatory properties by significantly lowering IL-6 expressions in vitro at 20 µM [91].

**Figure 17:**
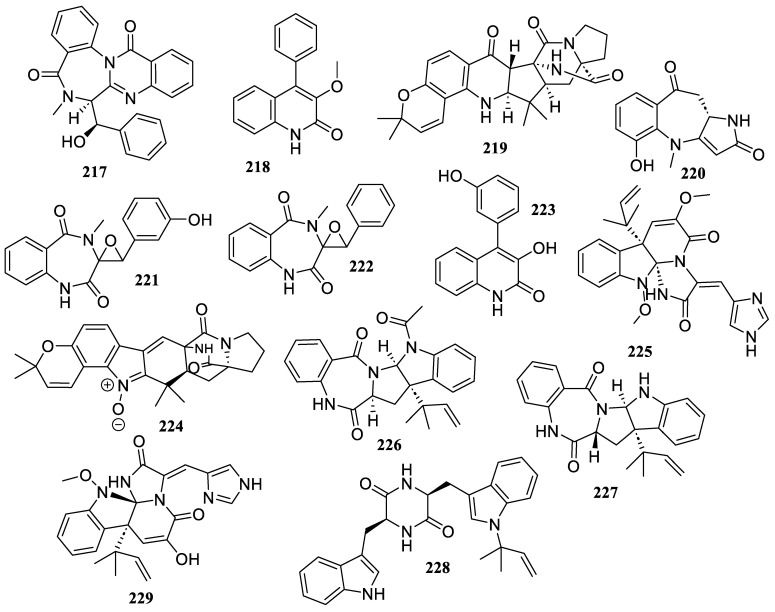
Chemical structures of alkaloids (**217**–**229**).

Two known compounds, benzomalvin E (**217**) and methylviridicatin (**218**) (Figure 17), were produced by the seawater-derived fungus *Metarhizium* sp. P2100. Both indicated anti-inflammatory activity against LPS-induced NO generation, with IC_50_ values of 37.1 µM and 37.5 µM, respectively [92].

A new compound, sclerotiamide J (**219**), was identified from the coral-derived fungus *Aspergillus sclerotiorum* LZDX-33-4. It prevented NLRP3 inflammasome-induced pyroptosis through the mitigation of mitochondrial damage, and greatly decreased its activation at a concentration of 10 µM [93].

The chemical investigation of the gorgonian coral-associated *Aspergillus candidus* CHNSCLM-0393 provided a pyrrolinone-fused 6/7/5 benzoazepine compound, (+)-asperazepanone B (**220**). It demonstrated strong anti-inflammatory activity by blocking the expression of TNF-*α* and IL-6 induced by LPS at a concentration of 0.1 μM [94].

Three compounds, cyclopenol (**221**), cyclopenin (**222**), and viridicatol (**223**), were isolated from the fungus *Aspergillus austroafricanus* Y32-2. They showed anti-inflammatory action in an inflammation-induced zebrafish model (ranging from 30 to 120 µg/mL) [75].

A chemical investigation of the fungus *Aspergillus* sp. YJ191021 yielded one new prenylated indole diketopiperazine, asperthrin A (**224**). It revealed strong anti-inflammatory activity with an IC_50_ value of 1.5 ± 0.2 µM in the human monocyte cell line (THP-1) generated by *Propionibacterium acnes* [95].

A known metabolite, oxaline (**225**), was obtained from cultures of *Penicillium oxalicum* CLC MF 05. It was found to suppress the overproduction of NO and PGE_2_, as well as the overexpression of iNOS and COX-2, in both LPS-stimulated BV2 and rat primary microglia with IC_50_ values between 8.8 ± 0.4 and 9.0 ± 0.5 µM [44].

Two compounds, *epi*-aszonalenin A (**226**) and aszonalenin (**227**), were obtained from the coral-derived fungus *Aspergillus terreus* C23-3. Both inhibited the phosphorylation of the MAPK and PI3K/AKT pathways, VEGF protein production, and LOX-1, triggered by ox-LDL at concentrations of 1–10 μM. Moreover, compound **227** inhibited the inflammatory factors (TNF-*α*, IL-1*β*, and IL-6) triggered by ox-LDL [96].

A known compound, cyclo (N^8^-(*α*, *α*-dimethylallyl)-l-Trp-l-Trp) (**228**), was isolated from the hydrothermal vent sediment-derived fungus *Penicillium* sp. LSH-3-1. It decreased the LPS-induced production of pro-inflammatory mediators, including NO, IL-6, and TNF-*α* at concentrations of 20 to 50 μM [97].

The chemical investigation of the deep-sea sediment-derived fungus *Penicillium chrysogenum* strain S003 yielded one known compound, meleag (**229**). It reduced the levels of IL-6 and IFN-*γ*, downregulated the expressions of the TLR4, TNF-*α*, and NF-*κ*B genes, and controlled the Nrf-2/HO-1 cascade [98].

### 2.4. Amides or Peptides

In total, 12 amides or peptides with anti-inflammatory activity were obtained from marine microorganisms during 2021–2023.

**Figure 18:**
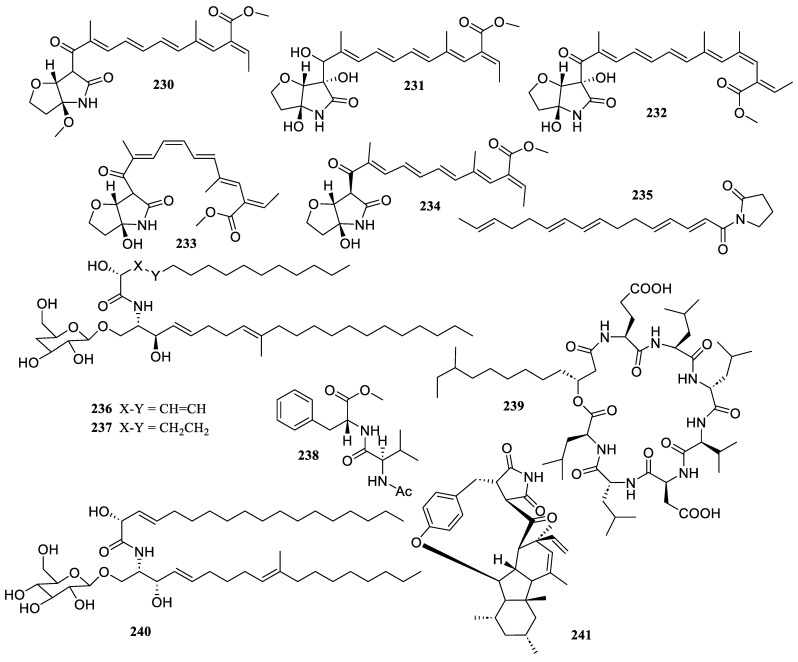
Chemical structures of amides and peptides (**230**–**241**).

Five known compounds, 3,5,7,9-undecatetraenoate (**230**), methyl (2*E*,3*E*,5*E*,7*E*,9*E*)-11-((3a*S*,6*S*,6a*R*)-3a,6-dihydroxy-5-oxohexahydro-2*H*-furo [3,2-b] pyrrol-6-yl)-2-ethylidene-11-hydroxy-4,10-dimethylundeca-3,5,7,9-tetraenoate (**231**), 4*Z*-lucilactaene (**232**), 8*Z*-lucilactaene (**233**), and lucilactaene (**234**) (Figure 18), were isolated from the fungus *Fusarium solani* 7227. They presented strong anti-inflammatory activity by preventing the formation of NO in RAW 264.7 cells stimulated by LPS, with IC_50_ values of 32.2 ± 5.7, 17.8 ± 4.9, 7.6 ± 2.0, 3.6 ± 2.2, and 8.4 ± 2.2 µM, respectively. Moreover, the polyunsaturated chain’s substitution group increased the anti-inflammatory properties [30].

A new compound, variotin B (**235**), was identified from the ethyl acetate extract of the shrimp-derived fungus *Aspergillus unguis* IV17-109. It indicated anti-inflammatory efficacy by blocking NO generation as well as the expression of iNOS and IL-6 with an IC_50_ value of 20.0 µM [99].

Two new cerebroside metabolites, hortacerebrosides A (**236**) and B (**237**), were discovered from the sponge-derived fungus *Hortaea werneckii* HN-YPG-2-5. Both showed a notable suppressive impact on the amount of NO generated by RAW 264.7 macrophages activated by LPS, with IC_50_ values of 5 and 7 μM, respectively [100].

One known compound, methyl acetyl-d-valyl-d-phenylalaninate (**238**), was isolated from the fungus *Penicillium* sp. LSH-3-1, which reduced the production of pro-inflammatory mediators, such as NO, IL-6, and TNF-*α*, at concentrations of 20 to 50 μM, when exposed to LPS [97].

Anteiso-C13-surfactin (IA-1) (**239**) was identified from the marine sediment-derived fungus *Bacillus amyloliquefaciens* strain IA-LB. It ameliorated the inflammatory damage to lung tissue by decreasing neutrophil infiltration, reducing elastase release and oxidative stress in endotoxemic mice at a concentration of 5 µM [101].

The chemical investigation of the sediment-derived fungus *Penicillium islandicum* yielded one known compound, flavuside B (**240**), which significantly reduced LDH release from LPS-induced HaCaT cells to the baseline NO level [102]. One known compound, GKK1032 B (**241**), was isolated from the deep-sea-derived *Penicillium citrinum* W17. It exhibited significant inhibitory effects on LPS-stimulated NO production in murine brain microglial BV2 cells in a dose–response manner with an IC_50_ value of 4.7 µM [72].

### 2.5. Steroids

In total, 11 steroids with anti-inflammatory activity were obtained from marine microorganisms during 2021–2023.

**Figure 19:**
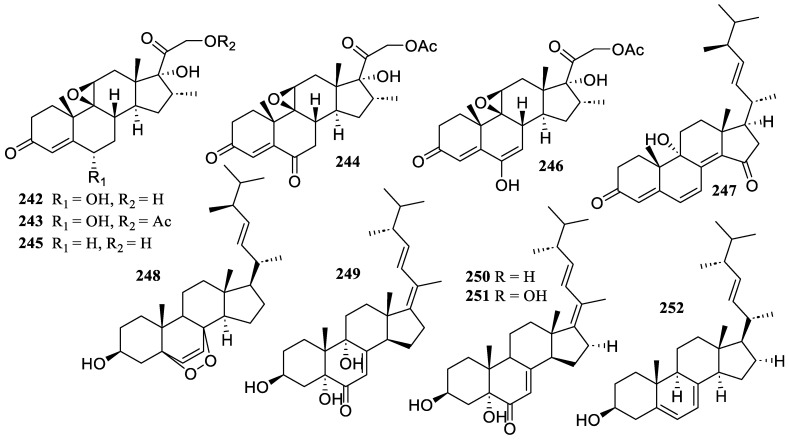
Chemical structures of steroids (**242**–**252**).

The fungus *Simplicillium lanosoniveum* SCSIO 41212 produced four new steroids derivatives, arthriniumsteroids A–D (**242**–**245**), and two known compounds, penicildione B (**246**) and ganodermaside D (**247**) (Figure 19). They displayed poor inhibitory abilities at a dosage of 40 µg/mL, with inhibitory rates ranging from 21.4% to 44.6% [103].

The chemical investigation of the seagrass *Enhalus acoroides*-associated fungus *Penicillium levitum* N33.2 yielded one known compound, ergosterol peroxide (**248**). It indicated an inhibitory effect on macrophages’ generation of NO, with an inhibition rate of 81.4 ± 1.4% at 25 mg/mL [104].

Three new ergostane-type sterols, aspersterols B–D (**249**–**251**), were isolated and identified from the shrimp-derived fungus *Aspergillus unguis* IV17-109. They inhibited LPS-induced NO generation with IC_50_ values of 19.5 ± 1.2, 11.6 ± 1.6, and 14.5 ± 1.5 µM, respectively [105].

One known compound, (22*E*, 24*R*)-ergosta-5,7,22-trien-3*β*-ol (**252**), was obtained from the fungus *Amorosia* sp. SCSIO 41026. It showed inhibitory effects on the overproduction of NO and pro-inflammatory cytokines in LPS-challenged RAW 264.7 macrophages without cytotoxicity at a concentration of 10 µM [56].

## 3. Conclusions

**Figure 20:**
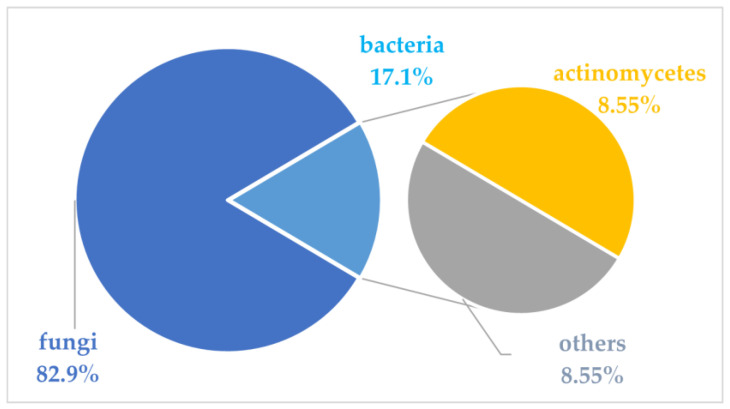
The sources of marine microbial anti-inflammatory natural products (2021–2023).

**Figure 21:**
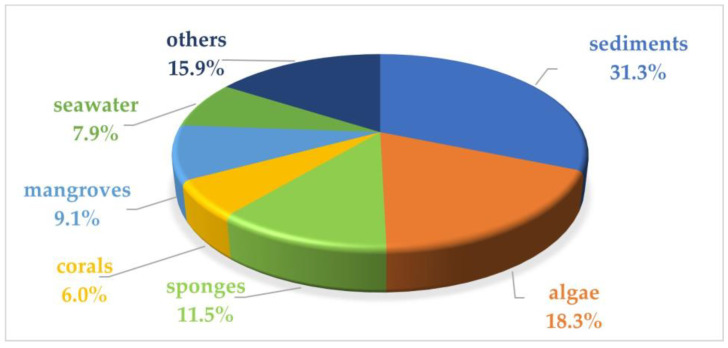
The habitat distribution of anti-inflammatory natural product-producing marine microorganisms.

**Figure 22:**
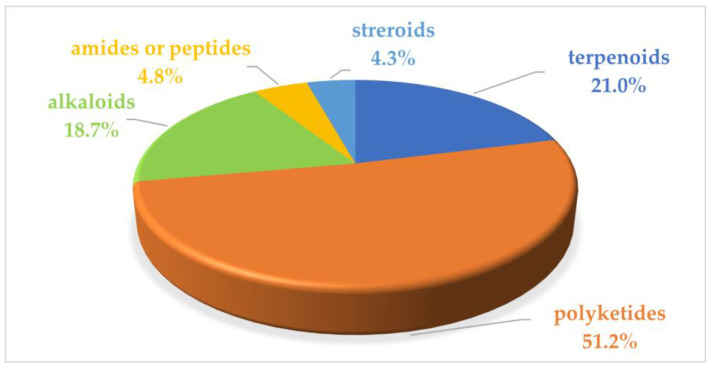
Structural types of marine microbial anti-inflammatory natural products (2021–2023).

This review summarizes the sources, chemical structures, and pharmacological properties of anti-inflammatory natural products reported from marine microorganisms in the past three years. A total of 252 natural products with anti-inflammatory activity were recently identified from marine microorganisms, while 51.2% of them were new compounds (Table S1). In addition, 82.9% of them were derived from marine fungi, while 17.1% of them were obtained from marine bacteria or marine actinomycetes (Figure 20). The reviewed marine microorganisms are derived from sediments (31.3%), algae (18.3%), sponges (11.5%), mangroves (9.1%), seawater (7.9%), corals (6.0%), and others (15.9%) (Figure 21). Moreover, the summarized compounds are structurally divided into polyketides (51.2%), terpenoids (21.0%), alkaloids (18.7%), amides or peptides (4.8%), and steroids (4.3%) (Figure 22). Related anti-inflammatory factors include NO, iNOS, NF-*κ*B, and PGE_2_. It is worth noting that the chemical structures of compounds **245**–**252** with significant anti-inflammatory activity show a high similarity to those of steroidal anti-inflammatory drugs like prednisone.

Marine microbial natural products are promising sources of anti-inflammatory lead compounds, especially those derived from marine fungi. New effective strategies for dereplication and prioritization to search for minor metabolites should be envisaged for the discovery of new natural compounds from marine microbial sources. Continuously optimizing the fermentation, strengthening the development of extraction and isolation, high-throughput screening, synthetic drug processes, and computer-assisted drug research technologies in the future will promote the mass production as well as the development of anti-inflammatory natural products into clinical agents. Through bioactivity-oriented approaches, diverse natural products with potent anti-inflammatory activity will be found and further structurally modified to improve their drug-forming properties, in order to develop them into anti-inflammatory candidate drugs.

Marine microbial natural products present promising applications in anti-inflammatory drug therapy. However, developing potential anti-inflammatory compounds into clinical agents still faces great challenges owing to their toxicity and selectivity. This review primarily elucidated the pharmacological mechanism of recently reported marine microbial anti-inflammatory natural products, which have attracted great interest and attention in marine microbial anti-inflammatory natural product research, and shed light on their value in the development of clinical anti-inflammatory drugs.

## Supplementary Materials

The following supporting information can be downloaded at: https://www.mdpi.com/article/10.3390/md22090424/s1, Table S1: Recently reported marine microbial natural products with anti-inflammatory activity (January 2021 through December 2023).

## Abbreviations

IC_50_	Half maximal inhibitory concentration
NF-*κ*B	Nuclear factor kappa-B
LPS	Lipopolysaccharide
NO	Nitric oxide
IL-6	Interleukine-6
TNF-*α*	TNF-*α*
