# Supplementary material for: Recent Advances in Anti-Inflammatory Compounds from Marine Microorganisms
Source: Mar Drugs. 2024 Sep 18;22(9):424. doi: 10.3390/md22090424 (PMC11433063; doi:10.3390/md22090424)
Supplement: Supplementary file 1 [file marinedrugs-22-00424-s001.zip › marinedrugs-3183098-supplementary.pdf]

Supplementary materials

Recent Advances in Anti-Inflammatory Compounds from Marine Microorganisms

Guihua Yang [1,†], Miaoping Lin [1,†], Kumaravel Kaliaperumal [2], Yaqi Lu [1], Xin Qi [1], Xiaodong Jiang [1], Xinya Xu [1], Chenghai Gao [1], Yonghong Liu [1] and Xiaowei Luo [1,*]

[1] Guangxi Key Laboratory of Marine Drugs, Institute of Marine Drugs, Guangxi University of Chinese Medicine, Nanning 530200, China
[2] Unit of Biomaterials Research, Department of Orthodontics, Saveetha Dental College and Hospitals, Saveetha Institute of Medical and Technical Sciences (SIMATS), Saveetha University, Chennai 600077, India
* Correspondence: luoxiaowei1991@126.com
† These authors contributed equally to this work.

Table S1. Recently reported marine microbial natural products with anti-inflammatory activity (January 2021 through December 2023)

Compounds	Producing Strains	Sources	Anti-inflammatory activity	Ref.
epiremisporines D–E (**1**–**2**)	*Penicillium citrinum* BCRC 09F458	waste water	fMLP-induced superoxide anion generation (human neutrophils) IC_{50}： 6.4 ± 0.4 (**1**), 8.3 ± 0.3 (**2**), 31.7 ± 2.5 (**3**), 33.5 ± 0.4 (**4**), 3.6 ± 0.6 (**5**), 2.7 ± 0.1 (**6**) μM	[24, 25]
epiremisporine G (**3**)				
penicitrinone H (**4**)				
epiremisporine B (**5**)				
penicitrinone A (**6**)				
epitetrahydrotrichodimer ether (**7**)	*Penicillium* sp. DM 815	the rhizosphere soil of *Hibiscus tiliaceus* Linn	LPS-induced the iNOS protein expression	[26]
tetrahydrotrichodimerol (**8**)				
4-carboxy-5-((1*Z*,3*E*)-1,3-heptadien1-yl)-1,3-benzenediol (**9**)	*Penicillium* sp. TW58-16	hydrothermal vent sediment	LPS-induced NO production (RAW 264.7)	[27]
decempyrone C (**10**)	*Fusarium decemcellulare*	seawater	LPS-induced NO production	[28]

decempyrone J (**11**)	SYSU-MS 6716		(RAW 264.7) IC_{50}: 22.4 ± 1.8 (**10**), 21.7 ± 1.1 (**11**) μM	
heterocornol T (**12**)<br>heterocornol X (**13**)	*Pestalotiopsis heterocornis* XWS 03F09	the sponge *Phakellia fusca*	LPS-induced the iNOS protein expression	[29]
fusarin K (**14**)	*Fusarium solani* 7227	seawater	LPS-induced NO production (RAW 264.7) IC_{50}: 21.9 ± 9.8 (**14**) μM	[30]
penicilazaphilone D (**15**)<br>penicilazaphilones F–G (**16–17**)<br>hypocrellone A (**18**)	*Penicillium sclerotiorum* E23Y-1A	sponge	LPS-induced NO production (BV2) IC_{50}: 34.8 ± 1.9 (**15**), 31.7 ± 1.5 (**16**), 34.5 ± 1.4 (**17**), 25.3 ± 2.2 (**18**) μM	[31]
saccharothrixin G (**19**)	*Saccharothrix* sp. D09	sediment	inhibited the production of NO IC_{50}: 28 (**19**) μM	[32]
(+)-terrein (**20**)<br>butyrolactone I (**21**)	*Aspergillus flavipes* (MTCC 5220)	the pneumatophores of mangrove plant *Acanthus ilicifolius*	exhibited IL-6 and TNF-*α* inhibition activity IC_{50}: 8.5 ± 0.7 (**20**) (IL-6), 15.8 ± 0.2 (**20**) (TNF-*α*), 12.0 ± 0.9 (**21**) (IL-6), 43.3 ± 0.8 μM (**21**) (TNF-*α*)	[33]
butyrolactone I (**21**)	*Aspergillus terreus* MT 273950	the annelide *Spirorbis* sp.	inhibited the release of neutrophil elastase IC_{50}: 2.3 ± 0.3 (**21**) μM	[34]
8a-*epi*-hypocrellone A (**22**)<br>hypocrellone A (**23**)<br>isochromophilone IV (**24**)	*Penicillium sclerotiorum* Al-27	alga *Grateloupia* sp.	inhibited the TNF-*α*-induced NF-*κ*B phosphorylation IC_{50}: 20 (**22–24**) μM	[35]
isochromophilone IV (**24**)<br>WB (**25**)	*Penicillium sclerotiorum* THSH–4 with *Penicillium sclerotiorum* ZJHJJ–18	a fresh flower of the marine mangrove *Aegiceras corniculatum*	LPS-induced NO production IC_{50}: 4.7–17.6 μM	[36]
5,9-dihydroxy-2,4,6,8,10-pentamethyldodeca-	*Aspergillus ochraceopetalif*	the alga *Hypnea pannosa*	LPS-induced NO production (RAW 264.7)	[37]

2,6,10-trienal (**26**)	*ormis* SCSIO 41020			
(3*R*, 4*S*) -(–)-4-hydroxymellein (**27**)				
(3*R*, 4*R*) -(–)-4-hydroxymellein (**28**)				
12-deacetylphomoxanthone A (**29**)	*Diaporthe* sp. SYSU-MS 4722	the ascidian *Styela plicata*	LPS-induced NO production (RAW 264.7) IC$_{50}$： 6.3–8.0 (**29–34**) μM	[38]
phomoxanthone A–B (**30–31**)				
dicerandrol B–C (**32–33**)				
deacetylphomoxanthone B (**34**)				
trypacidin (**35**)	*Talaromyces helicus* SCSIO 41311	a cold seep sediment	LPS-induced NO production (BV2) IC$_{50}$： 38.6 (**35**), 15.5 (**36**) μM	[39]
fumiquinone B (**36**)				
(4*S*)-4,10-dihydroxy-10-methyl-11-oxo-dodec-2-en-1,4-olide (**37**)	*Streptomyces* sp. 13G036	the marine sediment	inhibited the production of NO, TNF-*α* and IL-6 in LPS-stimulated macrophages	[40]
(4*S*)-4,10-dihydroxy-10 -methyl-undec-2-en-1,4-Olide (**38**)				
(4*S*)-4,10-dihydroxy-10-methyl-dodec-2-en-1, 4-olide (**39**)				
(4*S*,10*R*,11*S*)-4,11-dihydroxy-10-methyl-dodec-2-en-1,4-olide (**40**)				
(4*S*)-4-hydroxy-10-methyl-11-oxo-dodec-2-en-1,4-olide (**41**)				
(4*S*,10*S*,11*S*)-4,10,11-trihydroxy-10-methyl-dodec-2-en-1,4-olide (**42**)				
aspulvinone V (**43**)	*Aspergillus terreus* Thom	alga *Ulva lactuca* L.	LPS-induced NO production	[41]
(+)-terrein (**20**)				

butyrolactone I (**21**)	(Trichocomaceae) strain NTU243		(BV2)	
saadamysin (**44**)	*Aspergillus flavus* GXIMD 02503	a coral *Porites lutea*	inhibitory activities of NF-κB activation IC$_{50}$: 10.7 ± 1.3 (**44**) μM	[42]
pestaloketide A (**45**)	*Pestalotiopsis* sp. SWMU-WZ04-2	the sponge	LPS-induced NO production IC$_{50}$: 23.6 (**45**), 14.5 (**46**) μM	[43]
pestaloketide B (**46**)				
isorhodoptilometrin (**47**)	*Penicillium oxalicum* CLC-MF05	marine sponge	inhibited NO and PGE$_2$ overproduction and iNOS and COX-2 overexpression in both LPS-stimulated BV2 and rat primary microglia	[44]
5-hydroxy-7-(2′-hydroxypropyl)-2-methyl-chromone (**48**)				
penicillixanthone A (**49**)	*Aspergillus Sclerotiorum* SCSIO 41036	a soft coral BH6	LPS-induced NO production	[45]
streptoglycerides E–H (**50–53**)	*Streptomyces specialis* 208DD-067	a sediment sample	LPS-induced NO production (RAW 264.7) IC$_{50}$: 10.9 (**50**), 5.9 (**51**), 4.7 (**52**), 3.5 (**53**) μM	[46]
eschscholin B (**54**)	*Daldinia eschscholtzii* KBJYZ-1	the root of *Pluchea indica* Less.	LPS-induced NO production (RAW 264.7) IC$_{50}$: 19.3 (**54**), 12.9 (**55**) μM	[47]
daldilene A (**55**)				
amphichopyrones A–B (**56–57**)	*Amphichorda felina* SYSU-MS 7908	a marine ascidian	LPS-induced NO production (RAW 264.7) IC$_{50}$: 18.09 ± 4.83 (**56**), 7.18 ± 0.93 (**57**) μM	[48]
nectriapyrone (**58**)	*Diaporthe* sp. SYSU-MS 4722	the ascidian	LPS-induced NO production IC$_{50}$: 35.4 (**58**), 40.8 (**59**) μM	[49]
monodictyphenone (**59**)				
aspergillusidone H (**60**)	*Aspergillus unguis*	a coral *Pocillopora damicornis*	LPS-induced NF-κB activation (RAW 264.7)	[50]
aspergillusether J				

(**61**)	GXIMD 02505			
aspergillusether F (**62**)				
nornidulin (**63**)				
aspergillusidone B (**64**)				
guisinol (**65**)				
1-(2,6-dihydroxy-4-methoxy-3,5-dimethylphenyl)- 2-methylbutan-1-one (**66**)				
4,8-dihydroxy-6-methoxy-4,5-dimethyl-3-methyleneisochroman-1-one (**67**)	*Eutypella scoparia* HBU-91	collected from the Bohai Sea	LPS-induced NO production (RAW 264.7)	[51]
banksialactone A (**68**)				
streptinone (**69**)	*Streptomyces massiliensis* 213DD-128	a sediment sampl	suppressed the production of NO, PGE_2, and pro-inflammatory cytokines such as TNF-α, IL-6, and IL-1β, by inhibiting the TLR-mediated NF-κB	[52]
lithocarol F (**70**)	*Phomopsis lithocarpus* FS 508	a deep-sea sediment sample	LPS-induced NO production (RAW 264.7) IC_{50}: 22.8 (**70**), 27.2 (**71**), 24.1 (**72**) μM	[53]
isoprenylisobenzo-furan A (**71**)				
anhydromevalonolactone (**72**)				
stagonospones A–B (**73–74**)	*Stagonospora* sp. SYSU-MS 7888	a sponge	LPS-induced NO production IC_{50}： 3.6 ± 1.0 (**73**), 9.4 ± 1.8 (**74**), 21.9 ± 3.5 (**75**), 22.8 ± 3.9 (**76**) μM	[54]
stapyrone E (**75**)				
stapyrone G (**76**)				
penicilazaphilone N (**77**)	*Penicillium sclerotiorum* E23Y-1A	the marine sponge *Holoxea* sp.	LPS-induced NO production IC_{50}： 22.6 ± 3.0 (**77**) μM	[55]
chlomophenol A (**78**)	*Amorosia* sp. SCSIO 41026	the leaf of *Avicennia marina* (Forsk.)	possessed inhibitory effects against the excessive production of NO and pro-	[56]
7-chloro-3,4-dihydro-6,8-dihydroxy-3-				

methylisocoumarine (**79**)			inflammatory cytokines in LPS-challenged RAW 264.7 macrophages	
α-acetylorcinol (**80**)				
(*S*)-5,7-dichloro-6-methoxy-2-methyl-2,3-dihydrobenzofuran-4-carboxylic acid (**81**)				
5-chloro-6-hydroxymellein (**82**)				
3-methyl-6-hydroxy-8-methoxy-3,4-dihydroisocoumarin (**83**)				
kojic acid (**84**)				
orsaldechlorins A–B (**85–86**)	*Acremonium sclerotigenum* GXIMD 02501	coral-derived	LPS-induced NF-*κ*B activation (RAW 264.7)	[57]
ethyl orsellinate (**87**)				
5-chloroorsellinic acid (**88**)				
orcinol (**89**)				
O-methylorcinol (**90**)				
aryl bromide (**91**)				
ethyl 4-hydroxyphenylacetate (**92**)				
nectriatone C (**93**)				
diaporspchromanones B–C (**94–95**)	*Diaporthe* sp. XW12–1	the mangroves	LPS-induced NO production IC_{50}: 19.1 ± 3.6 (**94**), 9.6 ± 0.2 (**95**) μM	[58]
streptothiomycin E (**96**)	*Streptomyces* sp. DS-27	a rhizosphere sediment of coastal cordgrass *Spartina alterniflora*	LPS-induced NO production	[59]
S-methyl (4*R*,5*S*)-2,3-dimethyl-4- hydroxy-4-isopropyl-1-oxocyclopent-3-ene-5-carbothioate (**97**)				
suncheonosides E–F (**98–99**)	*Streptomyces* sp. ZSN 77	a sediment sample	inhibited LPS-induced NO production	[60]
suncheonoside J (**100**)				

S-Methyl 4-hydroxy-6-isopropyl-2-methoxy-3,5-dimethylbenzothioate (**101**)				
S-methyl 2,4-dihydroxy-6-isopropyl-3,5-dimethylbenzothioate (**102**)				
(4*S*,5*S*,6*S*,7*R*)-4-(3-chloro-1,2-dihydroxybutyl)-butyrolactone (**103**)	*Neofusicoccum parvum* Y2NBKZG 1016	the fruits of the mangrove plant *Sonneratia glauca*	LPS-induced NO production	[61]
talacyanol B (**104**)	*Talaromyces cyanescens*168 ST-51.1	the seaweed *Caulerpa* sp.	inhibited NO production and LPS-induced expression of COX-2 and iNOS in BV2 cells	[62]
eurothiocin A (**105**)				
asperpropanols A–D (**106–109**)	*Aspergillus puniceus* SRRC 2155	the deep-sea sediment	reduced NO, TNF-*α*, and IL-6 production	[63]
2,4-dihydroxy-6-((3*E*,5*E*)-nona-3,5-dien-1-yl)-benzoic acid (**110**)				
5-[(3*E*,5*E*)-3,5-nonadienyl]-1,3-benzenediol (**111**)				
chiayiflavans D–E (**112–113**)	*Isoptericola chiayiensis* BCRC 16888	mangrove soil	LPS-induced NO production (RAW 264.7) IC_{50}: 17.1 (**112**), 9.4 (**113**) μM	[64]
diaporpyrone A (**114**)	*Diaporthe* sp. QYM12	the mangrove	LPS-induced NO production (RAW 264.7) IC_{50}: 12.5 (**114**) μM	[65]
alternariol (**115**)	*Pleosporales* sp. SF-7343	moss	inhibited the secretion of interleukin-8 and -6 in TNF-*α*/interferon-*γ*-treated HaCaT cells	[66]
guhypoxylonol A (**116**)		the mangrove	against the production of NO	[67]

guhypoxylonols C–D (**117-118**)<br>hypoxylonol B (**119**)	*Aspergillus* sp. GXNU-Y45		IC50: 14.4 ± 0.1 (**116**), 18.0 ± 0.1 (**117**), 16.7 ± 0.2 (**118**), and 21.1 ± 0.1 (**119**) μM	
homogentisic acid (**120**)<br>methyl (2,5-dihydroxyphenyl) acetate (**121**)<br>3-chloro-2,5-dihydroxybenzyl alcohol (**122**)	*Aspergillus* sp. IMBC-FP2.05	the marine sponge	against NO overproduction IC50: 28.2 (**120**), 14.2 (**121**),41.8 (**122**) μM	[68]
penicillol B (**123**)	*Penicillium* sp. BJR-P2	the mangrove	LPS-induced NO production (RAW 264.7) IC50: 12.0 (**123**) μM	[69]
bisorbicillchaetone B (**124**)	*Penicillium* sp. SCSIO06868	sediment	LPS-induced NO production (RAW 264.7) IC50: 38.4 ± 3.3 (**124**) μM	[70]
ochrathinols A–B (**125–126**)	*Aspergillus ochraceopetaliformis* SCSIO 05702	soil	LPS-induced NO production (RAW 264.7)	[71]
(3*R**,4*S**)-6,8-dihydroxy-3,4,7-trimethylisocoumarin (**127**)<br>sclerotinin C (**128**)<br>asperbiphenyl (**129**)	*Penicillium* citrinum W17	sediment	LPS-induced NO production (BV2)	[72]
spiromaterpenes D–F (**130–132**)	*Spiromastix* sp. MCCC 3A00308	deep-sea sediment	LPS-induced NO production (BV2) IC50： 26.0 ± 2 (**130**), 9.0 ± 1 (**131**), 20.0 ± 1 (**132**) μM	[73]
decumbenone A (**133**)	*Aspergillus austroafricanus* Y32-2	seawater	sulfate-induced zebrafish inflammation model	[74]
paraconulones B–E (**134–137**)<br>paraconulone G (**138**)	*Paraconiothyrium sporulosum* DL-16	coastal sediment	LPS-induced NO production (BV2) IC50： 6.9 ± 2.6 (**134**), 7.7 ±	[75]

4-*epi*-microsphaeropsisin (**139**)			2.0 (**135**), 2.8 ± 0.5 (**136**), 8.1 ± 2.9 (**137**), 8.1 ± 3.5 (**138**), 4.6 ± 3.5 (**139**) μM	
eutypeterpenes B–C (**140–141**)	*Eutypella* sp. MCCC 3A00281	deep-sea sediment	LPS-induced NO production (RAW 264.7) IC50： 13.4 ± 0.8 (**140**), 16.8 ± 1.0 (**141**), 11.8 ± 1.0 (**142**), 8.6 ± 1.0 (**143**), 14.3 ± 1.1 (**144**), 11.5 ± 1.2 (**145**), 18.3 ± 1.0 (**146**), 17.1 ± 1.0 (**147**) μM	[76]
eutypeterpenes M–N (**142–143**)				
eutypeterpenes P–Q (**144–145**)				
eudesma-3-en-11,15-diol (**146**)				
eudesma-4-en-11,15-diol (**147**)				
nigerin (**148**)	*Aspergillus niger* 164117	marine sponge *Dysidea* sp.	LPS-induced NO production (RAW 264.7) IC50： 8.5 (**148**), 4.6 (**149**) μM	[77]
ochracene J (**149**)				
(2*R*,4*R*,5*S*,5a*R*,7*R*,9a*S*,10*S*)-10-(hydroxymethyl)-5,5a,8-trimethyl-3,4,5,5a,6,7-hexahydro-2,5-methanobenzo[*b*]oxepine-4,7,9a,10(2H)-tetraol (**150**)	*Trichoderma brevicompactum* NTU 439	*M. rosea* marine alga	LPS-induced NO production IC50： 10 (**150**), 10 (**151**), 10 (**152**), 10 (**153**), 10 (**154**), 5.2 ± 0.4 (**155**), 10 (**156**) μM	[78]
(2*S*,2'*R*,4'*R*,5'*S*,5a'*R*,9a'*R*)-8'-(hydroxymethyl)-5',5a'-dimethyl-2',3',4',5',5a',6',7',9a'-octahydrospiro[oxirane-2,10'-[2,5]methanobenzo[*b*]oxepin]-4'-ol (**151**)				
2*S*,2'*R*,4'*R*,5'*S*,5a'*R*,9a'*R*)-8'-(hydroxymethyl)-5',5a'-dimethyldecahydrospiro[oxirane-2,10'-[2,5] methanobenzo[*b*]oxepin]-4'-ol (**152**)				

trichoderminol (**153**)<br>trichodermarin A (**154**)<br>trichodermarin E (**155**)<br>trichodermol (**156**)				
ustusolates H–I (**157–158**)	*Aspergillus insuetus* SYSU 6925	a seagrass-derived	LPS-induced NO production (RAW 264.7) IC_{50}: 21.5 ± 1.1 (**157**), 32.6 ± 1.2 (**158**) μM	[79]
hazianol J (**159**)	*Trichoderma* sp. SCSIOW 21	the deep-sea sediment-derived	LPS-induced NO production (RAW 264.7)	[80]
libertellenone Z (**160**)<br>libertellenone A (**161**)<br>libertellenone C (**162**)	*Eutypella* sp. D-1	the soil	LPS-induced NO production (RAW 264.7)	[81]
peniscmeroterpenoid A (**163**)<br>peniscmeroterpenoid D (**164**)<br>peniscmeroterpenoid L (**165**)	*Penicillium sclerotiorum* GZU-XW03-2	the intestinal tract of the *Onchidium* sp.	LPS-induced NO production (RAW 264.7) IC_{50}: 26.6 ± 1.2 (**163**), 8.8 ± 1.2 (**164**), 48.1 ± 2.5 (**165**) μM	[82, 83]
soyasapogenols B1–B11 (**166–176**)	*Nonomuraea* sp. MYH522	a marine actinomycete	suppressing the STING/ TBK1/NF-κB pathway	[84]
decurrencyclic B (**177**)	*Turbinaria decurrens*	the algae	against COX-2 and 5-lipoxygenase IC_{50}: 14.0 (COX-2), 3.0(5-lipoxygenase)μM	[85]
aspermeroterpenes D–H (**178–182**)	*Aspergillus terreus* GZU-31-1	the intestinal tract of the *Onchidium* sp.	LPS-induced NO production (RAW 264.7) IC_{50}: 6.7 ± 0.8 (**178**), 29.6 ± 3.9 (**179**), 22.2 ± 0.9 (**180**), 25.9 ± 3.1 (**181**), 26.5 ± 1.0 (**182**) μM	[86]
aspechinulins B–C (**183–184**)<br>isoechinulins A–B (**185–186**)<br>neoechinulin B (**187**)<br>cryptoechinuline G	*Aspergillus* sp. FS 445	the deep-sea sediment	effected in LPS-induced mouse marcrophages RAW 264.7. IC_{50}: 20.0–90.0 (**183–188**) μM	[87]

(**188**)				
peniazaphilone A (**189**)	*Penicillium sclerotiorum* THSH–4 with *Penicillium sclerotiorum* ZJHJJ–18	a fresh flower of the marine mangrove *Aegiceras corniculatum*	LPS-induced NO production IC$_{50}$: 4.7–17.6 (**189–190**) μM	[36]
isochromophilone VI (**190**)				
fumigaclavine C (**191**)	*Talaromyces helicus* SCSIO 41311	a cold seep sediment	LPS-induced NO production (BV2) IC$_{50}$: 23.5 (**191**), 26.5 (**192**), 21.4 (**193**), 25.0 (**194**), 29.6 (**195**), 9.7 (**196**), 32.4 (**197**), 32.2 (**198**) μM	[39]
isotryptoquivaline F (**192**)				
fumiquinazoline F (**193**)				
12,13-dihydroxyfumitremorgin C (**194**)				
cyclotryprostatin B (**195**)				
azaspirofuran A (**196**)				
14-norpseurotin A (**197**)				
11-*O*-methylpseurotin A (**198**)				
asperorydine Q (**199**)	*Aspergillus flavus* GXIMD 02503	a coral *Porites lutea*	inhibitory activities of NF-κB activation IC$_{50}$: 14.1 ± 1.5 (**199**), 21.8 ± 1.9 (**200**), 8.6 ± 1.3 (**201**), 17.4 ± 1.7 (**202**), 11.3 ± 2.0 (**203**), 6.5 ± 1.4 (**204**) μM	[42]
asperorydine O (**200**)				
asperorydine J (**201**)				
speradine H (**202**)				
cyclopiamide A (**203**)				
pyrazinemethanol (**204**)				
terreusinones B–C (**205–206**)	*Aspergillus tamarii* MCCF102	a marine sponge sample	inhibited NO production in a dose-dependent manner	[88]
terreusinone (**207**)				
phenazostatin J (**208**)	*Cystobasidium laryngis* IV17-028	a deep sea sediment	LPS-induced NO production (BV2) IC$_{50}$: 0.3 (**208**) μM	[89]
lecanicilliumins A–B (**209–210**)	*Lecanicillium fusisporum*	sample sediment	againsted NF-κB production using LPS	[90]

lecanicilliumins E–G (**211**–**213**)	GXIMD 00542		induced RAW 264.7 cells (RAW 264.7) IC_{50}: 18.5 ± 1.2 (**209**), 25.8 ± 1.3 (**210**), 23.1 ± 1.3 (**211**), 24.7 ± 1.2 (**212**), 26.5 ± 1.1 (**213**) μM	
marinacarboline glucuronide (**214**)	*Actinoalloteichus cyanogriseus* LHW 52806	the marine sponge *Phakellia fusca*	decreased the expressions of IL-6	[91]
marinacarboline L (**215**)				
cyanogramide (**216**)				
benzomalvin E (**217**)	*Metarhizium* sp. P2100	seawater	LPS-induced NO production IC_{50}: 37.1 (**217**), 37.5 (**218**) μM	[92]
methylviridicatin (**218**)				
sclerotiamide J (**219**)	*Aspergillus sclerotiorum* LZDX-33-4	a gorgonian coral (LZDX-33)	inhibited NLRP3 inflammasome activation and blocked NLRP3 inflammasome-induced pyroptosis *via* amelioration of mitochondria damage	[93]
(+)-asperazepanone B (**220**)	*Aspergillus candidus* (CHNSCLM-0393)	the gorgonian coral-derived	inhibited LPS-induced expression of TNF-*α* and IL-6 displayed anti-inflammatory activity in a $CuSO_4$-induced zebrafish	[94]
cyclopenol (**221**)	*Aspergillus austroafricanus* Y32-2	seawater	inflammation model induced human monocyte cell line (THP-1)	[74]
cyclopenin (**222**)				
viridicatol (**223**)				
asperthrin A (**224**)	strain *Aspergillus* sp. YJ191021	sample soil		[95]
oxaline (**225**)	*Penicillium oxalicum* CLC-MF 05	marine sponge	inhibited NO and PGE_2 overproduction and iNOS and COX-2 overexpression in both LPS-stimulated BV2 and rat primary microglia	[44]
epi-aszonalenin A (**226**)	*Aspergillus terreus* C23-3	the coral *Pectinia paeonia*	ox-LDL-induced inflammatory factors (IL-6, IL-1*β*, and TNF-*α*)	[96]
aszonalenin (**227**)				
cyclo (N^8-(*α*, *α*-	*Penicillium*	hydrothermal	LPS-induced production	[97]

dimethylallyl)-L-Trp-L-Trp) (**228**)	sp. LSH-3-1	vents sediment	of pro-inflammatory mediators, including NO, IL-6 and TNF-α	
meleag (**229**)	*Penicillium chrysogenum* strain S003	deep-sea sediment	modulated the Nrf-2/HO-1 cascade, downregulated NF-κB, TLR4 and TNF-α gene expressions and lowered IL-6 and IFN-γ levels	[98]
undecatetraenoate (**230**)	*Fusarium solani* 7227	seawater	LPS-induced NO production (RAW 264.7) IC_{50}: 32.2 ± 5.7 (**230**), 17.8 ± 4.9 (**231**), 7.6 ± 2.0 (**232**), 3.6 ± 2.2 (**233**), 8.4 ± 2.2 (**234**) μM	[30]
methyl (2*E*,3*E*,5*E*,7*E*,9*E*)-11-((3a*S*,6*S*,6a*R*)-3a,6-dihydroxy-5-oxohexahydro-2H-furo[3,2-*b*] pyrrol-6-yl)-2-ethylidene-11-hydroxy-4,10-dimethylundeca-3,5,7,9-tetraenoate (**231**)				
4*Z*-lucilactaene (**232**)				
8*Z*-lucilactaene (**233**)				
lucilactaene (**234**)				
variotin B (**235**)	*Aspergillus* unguis IV17-109	a deep-sea shrimp sample	inhibited the production of NO and the expression of iNOS and IL-6 IC_{50}: 20.0 (**235**) μM	[99]
hortacerebrosides A–B (**236–237**)	*Hortaea werneckii* HN-YPG-2-5	the surface of sponges	LPS-induced NO production (RAW 264.7) IC_{50}: 7.0 (**236**), 5.0 (**237)** μM	[100]
methyl acetyl-*D*-valyl-*D*-phenylalaninate (**238**)	*Penicillium* sp. LSH-3-1	hydrothermal vents sediment	LPS-induced production of pro-inflammatory mediators, including NO, IL-6 and TNF-α	[97]
anteiso-C13-surfactin (IA-1) (**239**)	*Bacillus amyloliquefaciens* strain IA-LB	marine sediment	by decreasing neutrophil infiltration, reducing elastase release and oxidative stress in endotoxemic mice	[101]

flavuside B (**240**)	*Penicillium* is*landicum*	the sediment	treated of HaCaT cells with LPS	[102]
GKK1032 B (**241**)	*Penicillium citrinum* W17	the sediment	LPS-induced NO production (BV2) IC_{50}: 4.7 (**241**) μM	[72]
arthriniumsteroids A–D (**242–245**)	*Simplicillium lanosoniveum* SCSIO 41212	soft coral *Sinularia* sp.	LPS-induced NO production (RAW 264.7)	[103]
penicildione B (**246**)				
ganodermaside D (**247**)				
ergosterol peroxide (**248**)	*Penicillium levitum* N33.2	the leaf of seagrass *Enhalus acoroides*	inhibited of NO production in RAW 264.7 macrophages (RAW 264.7)	[104]
aspersterols B–D (**249–251**)	*Aspergillus unguis* IV17-109	*shrimp sampl*e	LPS-induced NO production IC_{50}: 19.5 ± 1.2 (**249**), 11.6 ± 1.6 (**250**), 14.5 ± 1.5 (**251**) μM	[105]
(22*E*, 24*R*)- ergosta-5,7,22-trien-3*β*-ol (**252**)	*Amorosia* sp. SCSIO 41026	the leaf of *Avicennia marina*	LPS-induced NO production (RAW 264.7)	[56]
